# Methyltransferase MGMT upregulation drives metastasis by activating epithelial-mesenchymal transition in KRAS mutant colon cancer

**DOI:** 10.1038/s41419-026-08858-z

**Published:** 2026-05-16

**Authors:** Gaixia Liu, Jiaqi Zhang, Qixin Li, Xinzhu Xue, Xiaolong Guo, Jing Han, Guanghui Wang, Feiyu Shi, Kexuan Wang, Yi Ding, Hong Wu, Chenhao Hu, Junjun She, Yinnan Chen

**Affiliations:** 1 Department of General Surgery, The First Affiliated Hospital of Xi’an Jiaotong University, Xi’an, Shaanxi 710061 China; 2 Center for Gut Microbiome Research, Med-X Institute, The First Affiliated Hospital of Xi’an Jiaotong University, Xi’an, Shaanxi 710061 China; 3 Department of High Talent, The First Affiliated Hospital of Xi’an Jiaotong University, Xi’an, Shaanxi 710061 China; 4 Hubei Province Key Laboratory of Precision Radiation Oncology, Wuhan, 430022 China

**Keywords:** Metastasis, Gastrointestinal cancer

## Abstract

Kirsten rat sarcoma (KRAS)-mutant colorectal cancer (CRC) is characterized by aggressive metastatic progression and profound therapeutic resistance. While direct inhibitors have emerged recently, their clinical application is often limited by rapid adaptive resistance, highlighting the urgent need to explore new therapeutic targets. Here, we identify O6-methylguanine-DNA methyltransferase (MGMT), a classical DNA repair enzyme, as a novel epigenetic facilitator of metastasis in KRAS-mutant CRC. Through integrated analyses of clinical cohorts and orthotopic metastasis models, we found that MGMT is upregulated in KRAS-mutant tumors and correlates with liver metastasis. Functionally, MGMT promotes epithelial-mesenchymal transition (EMT) and enhances metastatic capacity in vivo and in vitro. Mechanistically, we unveil a non-canonical function of MGMT in CRC: it interacts with histone H3 and reduces repressive H3K9me3 marks at the promoter of the epithelial-mesenchymal transition master regulator TWIST1, thereby activating its transcription. Importantly, we demonstrate that targeting MGMT sensitizes KRAS-mutant colorectal cancer to anti-EGFR therapy in preclinical models, providing a novel combinatorial strategy for this recalcitrant cancer subtype. Our study redefines MGMT as a multifaceted chromatin-modifying protein and establishes it as a promising therapeutic vulnerability to bypass resistance in KRAS-mutant CRC.

## Introduction

Colorectal cancer (CRC) is the third most common malignancy worldwide, accounting for nearly 8.5% of all cancer deaths [1]. CRC displays pronounced phenotypic heterogeneity and susceptibility to distant metastasis [2]. The Kirsten rat sarcoma (KRAS) oncogene is mutated in approximately 60% of metastatic CRC cases and is associated with resistance to receptor tyrosine kinase inhibitors, increased metastatic potential, poorer differentiation, and reduced survival [3]. Although research in molecular biology has improved our understanding of KRAS-mutation-driven CRC, including the approval of inhibitors targeting KRAS G12C, the rapid emergence of drug resistance limits long-term benefit for many patients with KRAS-mutant CRC [4]. Therefore, elucidating mechanisms that drive progression in KRAS-mutant CRC and identifying new therapeutic vulnerabilities remain imperative.

Imbalanced histone modifications can lead to tumorigenesis and development, and the loss of methylation of histone H3 residues has been reported as a marker of KRAS-mutant CRC [5, 6]. KRAS mutation increases histone H3 lysine 9 lactylation, which promotes CRC progression by upregulating the cholesterol transporter GRAMD1A expression. Targeting H3K9la or GRAMD1A may thus represent a promising therapeutic strategy for KRAS-mutant CRC [7]. Therefore, altered H3 modifications are non-negligible in promoting the malignant phenotypes of KRAS-mutant CRC. Identifying novel regulators that connect KRAS signaling to chromatin remodeling has important implications for understanding and treating KRAS mutant CRC. O6-methylguanine-DNA methyltransferase (MGMT) is a DNA repair enzyme that transfers methyl groups from O6-alkylguanine lesions to its active-site cysteine, thereby preserving genomic integrity [8]. Some studies have suggested an association between MGMT protein expression and mutations in the KRAS oncogene and the p53 tumor‑suppressor gene in CRC [9, 10]. However, other reports have found no specific correlation with this sequence change [11]. The expression of MGMT in cancers is regulated through various signaling pathways, including Wnt/β-catenin, NF‑κB, Hedgehog, PI3K/AKT/mTOR, and JAK/STAT [12]. Therefore, further investigations are required to clarify the abundance of MGMT in colorectal cancer and its relationship with clinicopathological and molecular features.

Here, using orthotopic CRC metastasis NOG mice models and integrated molecular analyses, we identify MGMT as a mediator of metastasis in KRAS-mutant CRC. Our comprehensive data indicate that in KRAS-mutant CRC, MGMT upregulation reduces H3K9me3 levels at the promoters of epithelial-mesenchymal transition (EMT) drivers and promotes TWIST1 transcription, thereby driving EMT and enhancing metastasis. Finally, we proved that targeting MGMT can sensitize KRAS-mutant CRC to anti-EGFR therapy.

## Materials and methods

### Study cohorts

Two independent CRC cohorts were analyzed. Cohort 1 comprised fresh-frozen tumor specimens from 88 patients who underwent surgical resection at the First Affiliated Hospital of Xi’an Jiaotong University (45.5% male). These tissues were immediately snap-frozen in liquid nitrogen and stored at −80 °C. The CRC tissue microarray (*n* = 40; 47.5% male) was also constructed. All cases were pathologically confirmed as colorectal adenocarcinoma, and patients provided informed consent. Cohort 2 was an integrated validation set assembled from three GEO datasets (GSE122246, GSE80606, and GSE209746). After normalization and batch correction, these datasets were merged for downstream analyses. We also conducted a single-center retrospective cohort study at the First Affiliated Hospital of Xi’an Jiaotong University(cohort 3). A total of 135 consecutive patients diagnosed with colorectal cancer between January 2020 and December 2021 were initially identified. All cases were histologically confirmed as colorectal adenocarcinoma. Clinicopathological data were retrieved from the hospital’s electronic medical record system. Baseline variables included age, sex, primary tumor location (colon or rectum), and pathological TNM stage (including pT and pN stages) at diagnosis. Inclusion criteria were: (1) pathologically confirmed colorectal adenocarcinoma; (2) confirmed KRAS mutation via molecular testing; (3) availability of complete clinicopathological and follow-up records; and (4) age ≥18 years at the time of diagnosis. Exclusion criteria included: (1) known hereditary CRC syndromes (e.g., Lynch syndrome or familial adenomatous polyposis); (2) a history of other malignancies; (3) multiple primary or recurrent CRC; (4) concurrent inflammatory bowel disease (IBD); or (5) insufficient clinical or survival data. Following this screening process, 56 KRAS-mutant CRC patients were enrolled in the final analysis. Survival status was monitored through outpatient clinical records and telephone interviews. The primary endpoint was overall survival (OS), defined as the interval from the date of initial diagnosis to either death from any cause or the date of last follow-up. The follow-up concluded in June 2025.

### Animal experiments

**Fig. 1:**
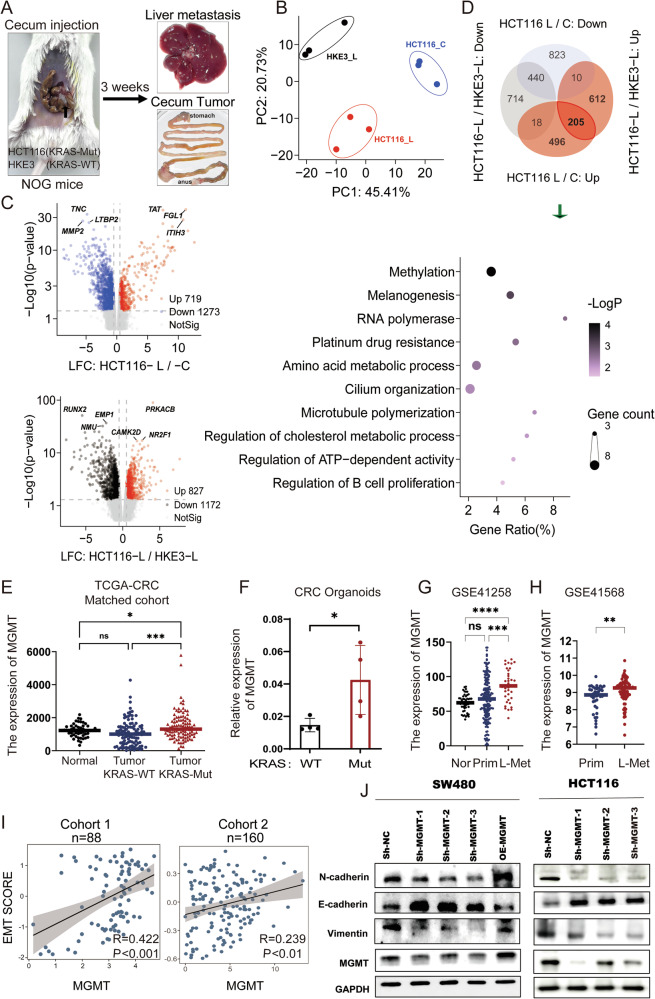
Increased MGMT was associated with liver metastasis in KRAS mutant colorectal cancer. **A** Schematic images of an in vivo orthotopic CRC metastatic model for identifying a metastasis-associated driver gene in KRAS mutant CRC. We leveraged two cell lines: HCT116 (KRAS mutant) and HKE3(KRAS wild type, derived from HCT116). **B** PCA of transcriptomic expression of the cecal original tumor (HCT116_C) and liver metastatic tumor (HCT116_L) from HCT116 groups, as well as the liver metastatic tumor from HKE3 (HKE3_L). **C** Volcano plots of differentially expressed genes in liver metastatic tumor relative to the cecal original tumor in the HCT116 group (top). Volcano plots of differentially expressed genes in liver metastatic tumor from the HCT116 group relative to the HKE3 group (bottom). **D** GO and KEGG pathway analysis based on the common 205 genes that were upregulated in HCT116 liver metastatic tumor, respectively, compared with HCT116 cecal original and HKE3 liver metastatic tumor. The top 10 pathways are showing. **E** Analysis of MGMT protein expression in normal tissue, KRAS mutant CRC, and KRAS wild type CRC in the TCGA cohort. **F** Relative expression of MGMT in KRAS mutant colon cancer organoids was compared with that of KRAS wild type organoids. **G**, **H** Analysis of MGMT protein expression in normal colon tissue (Nor), original CRC tissue (Prim), and liver metastatic cancer (L-Met) based on cohorts from GSE41258 and GSE41568 databases. **I** Correlation analysis of MGMT expression with epithelial-mesenchymal transition score in two CRC cohorts (*n* = 88 and *n* = 160, Spearman analysis). **J** Immunoblotting of EMT-related proteins (N-cadherin, E-cadherin, Vimentin) in ShRNA-MGMT (MGMT knockdown), OE-MGMT (MGMT overexpression) vs ShRNA-NC (normal control) CRC cells (KRAS mutant). Values are presented as mean ± SEM. *p < 0.05, **p < 0.01,***p < 0.001, ****p < 0.0001, determined by Mann–Whitney U test (**E**, **G**, **H**) and two-tailed Welch’s *t* test (**F**).

Male BALB/c or NOG mice aged 4–5 weeks were selected and housed under specific pathogen-free conditions before experimentation. Four distinct in vivo models were established: (1) Liver metastasis (spleen injection). 100 μL cell suspension containing 1 × 10^6^ CT26 or CT26-ShRNA-MGMT cells was injected into the spleens of BALB/c mice. Livers and tumors were harvested 15 days later. CT26 cells served as the control group (*n* = 5) and CT26-ShRNA-MGMT cells as the experimental group (*n* = 5). (2) Lung metastasis (tail vein): 100 μL cell suspension containing 1 × 10^6^ CT26-Luci cells or CT26-Luci-ShRNA-MGMT cells was injected into BALB/c mice via tail vein; Bioluminescence imaging (IVIS®Lumina III) was performed on day 15 after cell injection, and lungs and tumors were collected on day 21. CT26 cells served as the control group (*n* = 6) and CT26-ShRNA-MGMT cells as the experimental group (*n* = 6). (3) Orthotopic CRC liver metastasis: 5 × 10^6^ HCT116, HKE3, and HKE3-OE-MGMT cells were injected into the trigone of the mesentery of the cecum in NOG mice (Fig. 1A). Colon, liver, and cancer tissue were collected after 21 days. For transcriptomic profiling, primary tumors from HCT116 (*n* = 3), liver metastases from HCT116 (*n* = 3), and liver metastases from HKE3 (*n* = 3) were subjected to RNA sequencing analysis. (4) Subcutaneous tumor: 100 μL cell suspension containing 2 × 10^5^ CT26 or CT26-ShRNA-MGMT Cells was injected into the right iliac fossa of BALB/c mice, and the following tissues were harvested 15 days post-treatment. All mice bearing CT26/CT26-ShRNA-MGMT were blindly treated with Gefitinib (100 μg/g, gavage administration, MCE, #HY-50895) or O6-Benzylguanine (5ug/g, intraperitoneal injection, MCE, #HY-W002585) or combination therapy.

In the mouse experiments, researchers were blinded during model establishment, treatment assignment, and data analysis. Mice were randomly assigned to experimental and control groups using a computer-generated randomization sequence. All animal procedures were performed at Xi’an Jiaotong University under the approval of the Institutional Animal Care and Use Committee.

### RNA-sequencing analysis for orthotopic models

Paired 150-bp reads were adapter-trimmed and quality-filtered with fastp [13]. Processed reads were mapped to the human reference genome GRCh38 (GENCODE v47) using Salmon in selective alignment mode [14]. Transcript-level abundance estimates were aggregated to gene-level counts with Tximeta package [15]. DESeq2 was used to identify differentially expressed genes (pre-filter: >10 raw counts in ≥ 3 samples; threshold: |log2FC | > 0.5, *P* < 0.05) [16]. Enrichment analyses for GO Biological Processes and KEGG pathways were performed with Metascape (significance: hypergeometric *P* < 0.01 and minimum gene count ≥3) [17].

### Cell lines and culture

CRC cell lines (DLD1, HCT116, SW480, CT26, MC38, and SW837) were authenticated by short tandem repeat (STR) profiling. CT26 cells were cultured in RPMI-1640 + 10% fetal bovine serum (FBS, ExCell Bio Group, #FSD500); all other lines were maintained in Dulbecco’s Modified Eagle Medium (DMEM) + 10% FBS. The DMEM culture medium (KeyGEN BioTECH, #KGL1206-500) and the RPMI-1640 medium containing antibiotics (KeyGEN BioTECH, #KGL1501-500) were used. Cells were kept at 37 °C in 5% CO_2_.

### Generation of engineered cell lines

CRISPR/Cas9 was used to generate isogenic lines (MC38K) by editing KRAS as described. Taking MC38K as an example, this creates a DNA double-strand break (DSB) near the “GTC” sequence within the KRAS gene in MC38 cells (Table S1). A donor DNA template was provided to direct Homology-Directed Repair (HDR); monoclonal clones were selected and verified by PCR and sequencing.

### Stable transfection of knockdown and overexpression

The ShRNA-MGMT sequence was cloned into the pLKO.1 plasmid vector to construct the recombinant plasmid. The recombinant pLKO.1 plasmid, along with helper plasmids (pVSVG, pREV, and pGAG), was co-transfected into 293 T cells using Lipofectamine 3000 (Invitrogen, #L3000075). After 6 h, the culture medium was replaced. At 48 h post-transfection, supernatant was collected from 293 T cells by filtration through 0.45 μm filters, concentrated using PEG8000, and used to infect HCT116, DLD1, CT26, and SW480 cell lines. Selection was performed with puromycin (2 μg/mL) following infection.

The MGMT DNA coding sequence was PCR-amplified and inserted into the pLVX plasmid vector to construct the recombinant plasmid. The recombinant pLVX plasmid, along with packaging plasmids (psPAX2 and pMD2.G), was co-transfected into 293 T cells using Lipofectamine 3000. After 72 h, supernatant was collected from 293 T cells, concentrated with PEG8000, and used to infect HCT116, HKE3, and SW480 cell lines. Infected cells were selected with G-418 Sulfate (400 μg/mL). An identical approach was used for TWIST1 overexpression in CT26 cells, where the TWIST1 coding sequence was amplified and cloned into pLVX using OE-TWIST1 primers. The sequence primers were listed in Table S1.

### Migration and invasion

This study utilized commercial chambers to perform invasion assays on multiple cell types (Corning, #CLS354480). Using the HCT116 cell line as an example, 1 × 10^5^ cells were seeded into the upper chamber containing serum-free medium, while the lower chamber contained medium with 10% FBS. After 24 hours of incubation, cells in the lower chamber were washed with PBS, fixed with 4% paraformaldehyde, and stained with crystal violet. (For migration assays specifically, the upper chamber contained medium with 10% FBS). The number of cells was determined using ImageJ software. Five random microscopic fields were analyzed for each sample. The experiment for each cell type was independently repeated 3 times.

### CUT&Tag and CUT&Tag qPCR

CUT&Tag analysis was performed using the CUT&Tag Assay Kit (Vazyme Biotech Co., Ltd., #TD904-C1) in HCT116, HKE3, HKE3-OE-MGMT, DLD1, DSK8, and DLD1-Si-MGMT cell lines, following the manufacturer’s optimized protocol and preliminary experimental results. 1 × 10^5^ Cells were collected by centrifugation at 600 × *g* for 5 min at room temperature and washed with Wash Buffer at an identical centrifugal force. Washed pellets were then resuspended in 100 μL Wash Buffer (20 mM HEPES pH 7.5, 150 mM NaCl, 0.5 mM spermidine, Roche Complete Protease Inhibitor EDTA-Free) per sample, transferred to activated ConA Beads Pro in 8-strip tubes, and incubated for 10 min at room temperature. Pre-chilled Antibody Buffer (50 μL) containing primary antibody (2 μL H3K9me3/IgG) was added to each sample for overnight incubation at 4 °C. Following incubation, 8-strip tubes were placed on a magnetic stand until solution clarification, then supernatant was removed, and secondary antibody (1:100 dilution, Vazyme, #Ab207) was added for 30-min incubation at room temperature with rotation. Subsequent washes were performed using Dig-Wash Buffer. pA/G-Tnp Pro diluted in Dig-300 Buffer was added to each sample, which was incubated with rotation at room temperature for 1 hour, followed by washing with Dig-300 Buffer. Subsequently, 50 μL of diluted TTBL was added to each sample, and the samples were incubated at 37 °C for 60 min in a PCR machine. Subsequently, 10% SDS and an appropriate amount of DNA Spike-in were added to each sample, mixed by gentle inversion, and incubated at 55 °C for 10 min. After magnetic stand separation, the supernatant was carefully collected. For DNA extraction, pre-treated DNA Extract Beads Pro were added to the samples and incubated at room temperature for 20 min. Samples were placed on a magnetic stand to remove the supernatant, then 200 μL of 1 × B&W Buffer was added and incubated at room temperature for 30 s. After repeated supernatant removal, tubes were left uncapped at room temperature until complete liquid evaporation. DNA Extract Beads Pro were then resuspended in 15 μL ddH_2_O. Library amplification and PCR product purification were performed, followed by CUT&Tag product sequencing. Before sequencing, library quality testing was conducted, and the results showed that we had established a high-quality library (Fig. S1). Stop buffer (Vazyme, #TD904-C1) was added to the DNA Extract Beads Pro, and the mixture was incubated at 95 °C for 5 min. The supernatant rich in DNA can be used for qPCR detection. The primer sequences for TWIST1 are listed in Table S2.

### Cut&Tag analysis

Sequencing data were processed using Cutadapt (v10.14806/ej.17.1.200) to remove adapter sequences, reads containing ambiguous bases (≥5% N-content), and low-quality regions (Phred score <20). Sequence alignment against the human reference genome (GRCh38) was performed using Bowtie2 [18] with default parameters, followed by rigorous exclusion of mitochondrial genome-mapped reads to mitigate potential nuclear DNA contamination. Genome-wide enrichment regions were identified using MACS2 [19] under stringent parameters (-q 0.05 –keep-dup all), defining peak coordinates, summit positions, and genomic distribution patterns. The resultant peaks were functionally annotated using ChIPseeker [20] to determine their associations with transcriptional start sites and gene bodies. The transcriptional start sites and gene bodies were derived from the GENCODE release 47 annotations, with promoter regions defined as the ± 5 kb regions surrounding each transcription start site. Genomic landscape visualization was performed using Integrative Genomics Viewer 21 (IGV), enabling a comprehensive evaluation of peak distributions across all samples. We performed CUT&Tag-seq in HCT116 and derived cells, and CUT&Tag-RNA in HCT116, DLD1, and their derived cells.

### Organoid culture

Tumor samples were collected from CRC patients who underwent surgical resection at the First Affiliated Hospital of Xi’an Jiaotong University, China. Cases were collected based on precise pathological diagnosis and patient consent. Tumors were cut into pieces, and two parts were processed for organoid culture and DNA isolation. DNA was isolated for polymerase chain reaction (PCR) amplification, with subsequent sequencing to determine KRAS mutational status. The tumor tissues for organoid culture were promptly treated with antibiotics (incubated at 4 °C for at least 1 h). And the tissue was cut into smaller pieces and incubated with Collagenase II (1.5 mg/mL), Hyaluronidase (20 μg/mL), and Ly27632 (10 μM) for 40 min at 37 °C while shaking. After enzymatic digestion to release tumor stem cells, they were washed with PBS. The cell suspension was filtered through 100 μm cell strainers, then embedded in Matrigel (Corning, #356231). The mixture was dropped onto a pre-warmed 6-well plate. Once the Matrigel solidified, organoid-specific medium (IntestiCult™ Organoid Growth Medium, #06010) was added. The medium was changed every 3 days, and the organoids underwent the first division approximately on day 10. Furthermore, RNA was extracted for quantitative real-time PCR (qPCR) analysis to assess MGMT expression levels. The primer sequence is listed in Table S2.

### Protein extraction and western blot analysis

Cellular proteins were extracted using RIPA buffer containing protease and phosphatase inhibitors (Beyotime, #P0013C). For tissue proteins, samples were homogenized using a tissue grinder during lysis to ensure complete disruption before identical processing. Cells were lysed via sonication (30 s on/30 s off for 10 cycles) followed by centrifugation at 21,000 × *g* for 5 min at 4 °C. The supernatant was collected for BCA quantification (Beyotime, #P0010). Protein samples were denatured in SDS loading buffer at 95 °C for 5 min, separated by SDS-PAGE, and transferred to PVDF membranes. Membranes were blocked with 5% non-fat milk in TBST for 1.5 h at room temperature, then incubated overnight with primary antibodies (diluted in 5% milk/TBST). After three TBST washes, membranes were incubated with secondary antibodies for 1 h at room temperature. Following three additional TBST washes, protein bands were visualized using a chemiluminescent substrate (Biosharp, #BL520B) for imaging. Antibodies used in this study are listed in Table S3. All full and uncropped western blots were uploaded as Supplementary Material-uncropped western blots.

### Immunohistochemistry and immunofluorescence

The correlation between the level of H3K9me3 and MGMT expression was assessed by immunohistochemistry and immunofluorescence in CRC tissue microarray, as well as paraffin-embedded sections of mice CT26-subcutaneous tumor. Staining for H3K9me3 (Cell Signaling Technology, #9751S) was performed on paraffin-embedded sections of CRC tissue microarray. A total of 40 tumor samples from CRC patients were collected, and the expression of MGMT was confirmed according to RNA-Seq data. All subjects signed the informed consent forms before the sample collection. The primary antibodies used for immunofluorescence analysis in paraffin-embedded sections of mice CT26-subcutaneous tumor were H3K9me3 (Cell Signaling Technology, 9751S), MGMT (Proteintech, #17195-1-AP), and DAPI (Servicebio, #G1012).

Moreover, Ki-67 staining by immunohistochemistry on paraffin-embedded sections of liver metastases were performed to compare cell proliferation between controls and *MGMT* knockdown groups. Ki-67 staining by immunohistochemistry and TUNEL staining by immunofluorescence on sections of the CT26-subcutaneous tumor from mice that accepted therapy were performed to evaluate the therapeutic efficacy of targeting *MGMT* in combination with an anti-EGFR drug.

Images were captured using Case Viewer. The level of H3K9me3 was determined in tumor tissue using ImageJ software. Five random microscopic fields were analyzed for each sample.

### Cell viability assay

Cell viability was assessed using the CCK-8 assay (Biosharp, #BS350A). HCT116, HCT116-ShRNA-MGMT, SW480, and SW480-ShRNA-MGMT cells were seeded in 96-well plates at a density of 5 × 10^3^ cells/well and allowed to adhere overnight. Following the establishment of control groups, experimental groups were treated with a gradient concentration of Gefitinib for 48 h. Subsequently, 10 μL of CCK-8 solution was added to each well, followed by incubation in a cell culture incubator for 1–4 h. Absorbance was measured at 450 nm using a micro-plate reader, and dose-response curves were generated based on the absorbance readings.

### Flow cytometry analysis

HCT116 and SW480 cell lines, along with their MGMT-knockdown counterparts, were treated with 10 μM/20 μM Gefitinib for 48 h. Apoptosis analysis was then performed using flow cytometry according to the manufacturer’s instructions of the apoptosis detection kit (KeyGEN BioTECH, #KGA1102-100). Briefly, 5 × 10^5^ cells were harvested using trypsin without EDTA, re-suspended in 500 μL Binding Buffer containing 5 μL Annexin V-FITC and 5 μL Propidium Iodide, and incubated in the dark at room temperature for 10 min. Flow cytometric analysis was conducted within 1 h.

### Co-immunoprecipitation (Co-IP) and mass spectrometric analysis

HCT116 and SW480 cells were harvested, washed, and resuspended in lysis buffer [50 mM Tris-HCl (pH 8.0); 120 mM NaCl; 5 mM EDTA; 1% NP-40; 10% glycerol; Roche Complete Protease Inhibitor EDTA-Free; 2 mM Na3VO4] and kept on ice for 20 min. Cell extracts were sonicated with Bioruptor Plus (Biosense) at 4 °C, clarified by centrifugation, and proteins immobilized by binding to anti-HA (Sigma-Aldrich, #H6908) and IgG (Proteintech, #B900610) with gentle shaking overnight at 4 °C. Beads were washed and proteins recovered directly in SDS-PAGE sample buffer. Two independent experiments were performed and were directly subjected to mass spectrometry analysis. Base Peak of HA group and IgG group were shown in Fig. S1B. MGMT (Proteintech, #17195-1-AP) and H3 (Proteintech, #17168-1-AP) were used for western blot analysis. Sequencing data are provided in Supplementary Data 1.

### Co-IP analysis

Protein-to-gene identifier mapping of two independent experimental replicates was performed using the UniProt database [21]. Subsequent functional pathway enrichment analysis, encompassing Gene Ontology Biological Processes, KEGG Pathway, WikiPathways, and Reactome Gene Sets, was executed on the overlapping gene set identified through comparative analysis using Metascape [17], with terms meeting a hypergeometric *p* value < 0.01 and a minimum gene count >5 considered statistically significant.

### Global DNA 5mC methylation in the CRC cells

Global DNA methylation levels were quantified using the Global DNA Methylation Assay Kit (Abcam, #ab233486). Genomic DNA was extracted from parental HCT116 cells, MGMT-knockdown and MGMT-overexpression HCT116 (Identical procedures were performed for the SW480 cell line), and ensuring relative purity (260/280 ratio >1.6) for each sample. According to the manufacturer’s protocol, 100 μL of binding solution and 100 ng of sample DNA were added to each well, and the mixture was incubated at 37 °C for 60 min. After removal of the binding solution and washing, 50 μL of 5-mC detection antibody solution was added and incubated at room temperature for 50 min. After removing the detection solution and washing, the development solution was added. The reaction was terminated with stop solution after the 5% positive control showed deep blue color development, and absorbance was measured at 450 nm within 2–15 min using a microplate reader. Meanwhile, the standard curve was plotted, and the concentration of 5mC Methylation was compared among different groups.

### Calculation of EMT activity and statistical analysis

The EMT pathway activity for each sample in both cohorts was quantified using Gene Set Variation Analysis (GSVA). The gene set representing EMT was obtained from a previously published study (PubMed ID: 29920274) [22], which provided a well-defined list of EMT-related genes. The association between the computed EMT enrichment scores and *MGMT* gene expression levels was assessed in both cohorts using correlation analysis. Statistical significance was determined using Spearman’s correlation coefficient, selected because the data were non-normal. All analyses were performed in R (version 4.2.0) using appropriate bioinformatics packages.

## Results

### KRAS mutant CRC exhibited increased MGMT that was associated with liver metastasis

To discover key factors promoting liver metastasis in KRAS-mutant CRC, we used an in vivo orthotopic CRC metastatic model (Fig. 1A). HCT116 (mutant KRAS G13D) and HKE3 (mutant KRAS-wild type, derived from HCT116 by CRISPR/Cas9 editing, Fig. S2A) were implanted into the cecal serosa. We performed RNA-seq analysis of primary HCT116 tumors (HCT116_C), HCT116 liver metastases (HCT116_L), and HKE3 liver metastases (HKE3_L) (Supplementary data 2–5). Principal component analysis (PCA) showed significant differences in gene expression (DGE) characteristics among the HCT116_C, HCT116_L and HKE3_L groups (Fig. 1B). Comparing HCT116_L to HCT116_C identified 719 up-regulated genes, and comparing HCT116_L to HKE3_L identified 827 up-regulated genes (Fig. 1C). Based on shared 205 DEGs, we performed KEGG analysis and results suggested that *Methylation pathway* was enriched in HCT116 liver metastatic tumor compared with HKE3 liver metastatic and HCT116 colon primary tumor (Fig. 1D). Methylation affects tumor biological behavior, including metastasis, by influencing gene expression [23]. We further compared the expression of eight genes involved in the *methylation pathway* in gender- and age-matched (Normal: KRAS mutant CRC: KRAS wild Type=1:2:2) KRAS mutant and KRAS wild-type CRC cohort from TCGA (Table S4, Fig. S2B). Finally, we identified that MGMT was significantly increased in KRAS-mutant CRC, as determined in matched CRC organoids (Fig. 1E, F, Fig. S2C). Meanwhile, liver metastatic tumors displayed higher MGMT expression than primary CRC and normal colon in two independent cohorts (Fig. 1G, H). Higher MGMT levels were associated with poorer survival and more advanced clinical stages in KRAS-mutant CRC patients (Fig. S2D, Table S5).

Moreover, we established two independent CRC cohorts and found that high MGMT expression was associated with a mesenchymal state in CRC, as evidenced by a higher epithelial-mesenchymal transition score (Fig. 1I, Table S6). We established MGMT knockdown and overexpression cell lines from HCT116 and SW480 (mutant KRAS G12V) cells. Western blot analysis revealed decreased N-cadherin, Vimentin, and increased E-cadherin in CRC cell lines with lower MGMT. Consistently, increased N-cadherin and Vimentin, and decreased E-cadherin were observed in the overexpression of MGMT in SW480 compared with the normal control (Fig. 1J). These findings suggested that increased MGMT expression is positively correlated with KRAS mutations and liver metastasis in CRC patients.

### MGMT promotes CRC migration and invasion by regulating epithelial-mesenchymal transition

**Fig. 2:**
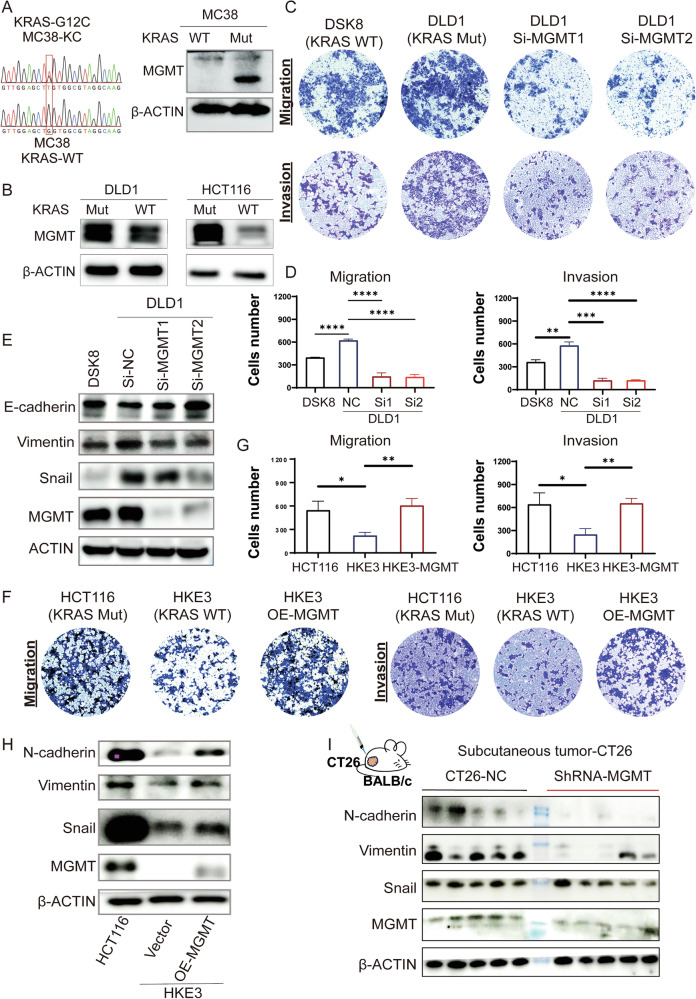
MGMT promotes CRC migration and invasion by regulating epithelial-mesenchymal transition. **A** Western blot analysis showing MGMT levels in KRAS mutant cells (DLD1 and HCT116) and their KRAS wild-type counterparts (DSK8 and HKE3). **B** MC38K with KRAS G12C mutant deriving from MC38 was generated, and the expression of MGMT was compared. **C**, **D** Transwell migration and Matrigel invasion assays in DLD1 and DSK8 cells treated with SiRNA-NC (normal control) and SiRNA-MGMT (MGMT knockdown). Quantification of migrated/invaded cells (*n* = 3 independent experiments). **E** Immunoblotting of EMT proteins (E-cadherin, Vimentin, Snail) and MGMT in DLD1 and DSK8 cells treated with SiRNA-NC and SiRNA-MGMT. **F**, **G** Transwell migration and Matrigel invasion assays in HCT116, HKE3, and HKE3-OE-MGMT (overexpression of MGMT with a plasmid in HKE3). Quantification of migrated/invaded cells (*n* = 3 independent experiments). **H** Immunoblotting of EMT-related proteins (N-cadherin, Vimentin, Snail) and MGMT in HCT116, HKE3, and HKE3-OE-MGMT. **I** Western blot analysis showing EMT-related proteins (N-cadherin, Vimentin, Snail) and MGMT in tumor tissue from mice bearing subcutaneous CT26/CT26-ShRNA-MGMT. Values are presented as mean ± SEM. *P < 0.05, **p < 0.01,***p < 0.001, ****p < 0.0001, determined by one-way ANOVA.

To further determine the functional impact of MGMT on tumor metastasis, we generated additional isogenic pairs (DLD1/DSK8, MC38/MC38K). The DSK8 cell lines (KRAS wild type), which were derived from DLD1(mutant KRAS G13D) through CRISPR/Cas9 (Fig. S2A), and the MC38K cell line (mutant KRAS G12C), which was derived from MC38 (KRAS wild type) (Fig. 2A). Firstly, the expression of MGMT was compared in HCT116/HKE3, DLD1/DSK8, and MC38/MC38K cell lines. Western blot shows a significant increase in MGMT expression in CRC cells with KRAS mutation (HCT116, DLD1, MC38K, Fig. 2A, B). Meanwhile, DSK8 showed a significantly decreased migration and invasion ability compared to DLD1. Consistently, migration and invasion capacity were significantly impaired in DLD1 with SiRNA-mediated MGMT knockdown (Fig. 2C, D). Moreover, MGMT overexpression in HKE3 restored its migration and invasion capacity compared with HCT116 (Fig. 2F, G). In accordance with these results, the expression of EMT representative markers (N-cadherin, Vimentin, E-cadherin, Snail1) changed concordantly with KRAS status or MGMT expression (Fig. 2E, H). Additionally, subcutaneously implanted CT26 (mutant KRAS G12D) tumors and MGMT-knockdown CT26 tumors were harvested to compare EMT marker expression. Decreased N-cadherin, Vimentin, and Snail1 in MGMT-knockdown CT26 tumors were validated (Fig. 2I). Altogether, these findings demonstrate that MGMT induces EMT and plays a significant role in the migration and invasion of KRAS mutant colorectal cancer.

### MGMT enhances CRC metastasis in vivo

**Fig. 3:**
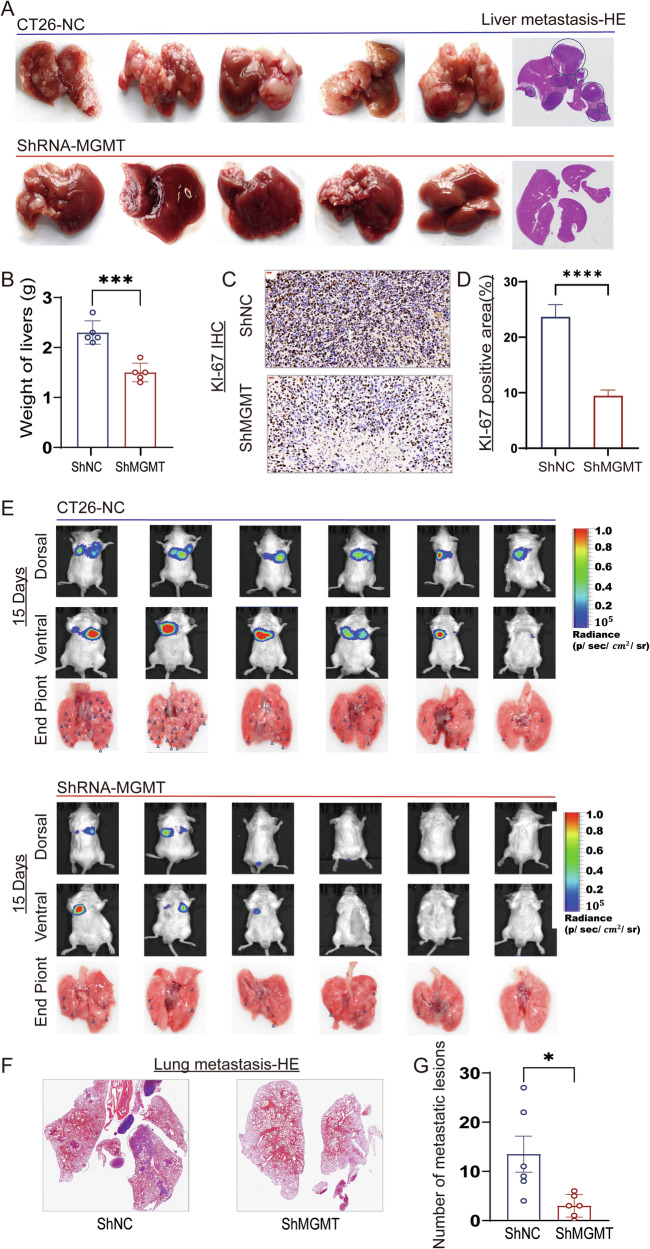
MGMT enhances CRC metastasis in vivo. **A** Liver metastasis mouse model was established by spleen injection, and MGMT knockdown inhibited liver metastasis (*n* = 5 mice per group). Representative images of H&E staining of liver tissues (right). **B** Quantification of the weight of mouse livers from MGMT knockdown groups and normal control groups. **C**, **D** Representative images and quantitative IHC analysis of Ki-67 in metastatic nodules of the liver (Scale bar: 20 μm). **E** Lung metastasis mice model was established by vein injection, and MGMT knockdown inhibited lung metastasis (*n* = 6 mice per group). Tumor growth in mice was observed using in vivo imaging system (IVIS) at 15 days post-injection (top). Representative images of the nude mouse lungs (bottom). **F** Representative images of H&E staining of lung tissues. **G** Quantification of the number of metastatic nodules on the surface of mouse lungs. Values are presented as mean ± SEM. *P < 0.05, ***p < 0.001, ****p < 0.0001, determined by two-tailed Welch’s *t* test.

To address the significance of these in vitro observations, we established mouse models by injecting CT26 cells knocked down for MGMT into the spleen or by tail vein injection to observe liver and lung metastasis. Our results showed that the MGMT knockdown group exhibited decreased liver metastasis, as evidenced by fewer metastatic nodules and lighter liver weight. Tumor metastatic nodules were determined by H&E stain (Fig. 3A, B). Furthermore, liver metastatic nodules in the MGMT knockdown group showed a lower Ki-67 level than those in the normal control group (Fig. 3C, D). In vivo imaging results of small animals showed that the MGMT knockdown group exhibited a significant decrease in lung metastasis after 15 days of tail vein injection. Notably, the MGMT knockdown group showed 83.3% (5/6) lung metastasis, compared with 100% (6/6) in the normal control group at the endpoint (21 days after cell injection). The MGMT knockdown group had fewer lung metastatic lesions, as determined by H&E stain (Fig. 3E–G).

**Fig. 4:**
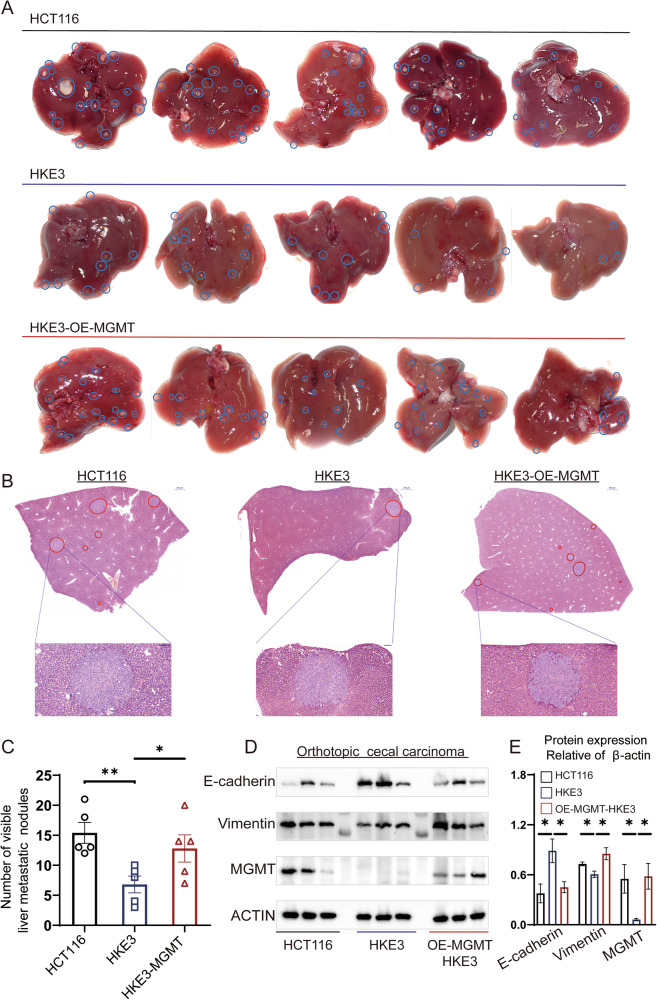
MGMT promotes CRC metastasis in the CRC orthotopic liver metastasis model. **A**, **B** Representative images of gross inspection and H&E staining of liver metastatic tumors from the CRC orthotopic tumor mice (*n* = 5 per group, Scale bar: 500 μm). **C** Quantification of the number of metastatic nodules on the surface of mice’s livers. **D**, **E** Immunoblotting of EMT-related proteins (E-cadherin, Vimentin) and MGMT in orthotopic cecal tumor from HCT116, HKE3, and HKE3-OE-MGMT groups. Densitometry quantification. Values are presented as mean ± SEM. *P < 0.05, **p < 0.01, determined by one-way ANOVA.

To extend this observation to another model, we used a CRC orthotopic liver metastasis model. HCT116, HKE3, and HKE3-OE-MGMT cells were implanted into the cecum of NOG mice, respectively (Fig. S3). We observed that HKE3 with wild-type KRAS showed less liver metastasis, compared to HCT116 with mutant *KRAS* (Fig. 4A). However, overexpressing MGMT in HKE3 significantly increased liver metastasis (Fig. 4A–C). Importantly, EMT was induced by MGMT, as seen by decreased Vimentin and increased E-cadherin in HKE3. HKE3-OE-MGMT showed similar expression of Vimentin and E-cadherin to HCT116 (Fig. 4D, E). Taken together, these in vivo data supported that MGMT promoted metastasis of KRAS mutant CRC through inducing EMT.

### MGMT interacts with histone H3 and downregulates H3K9me3

**Fig. 5:**
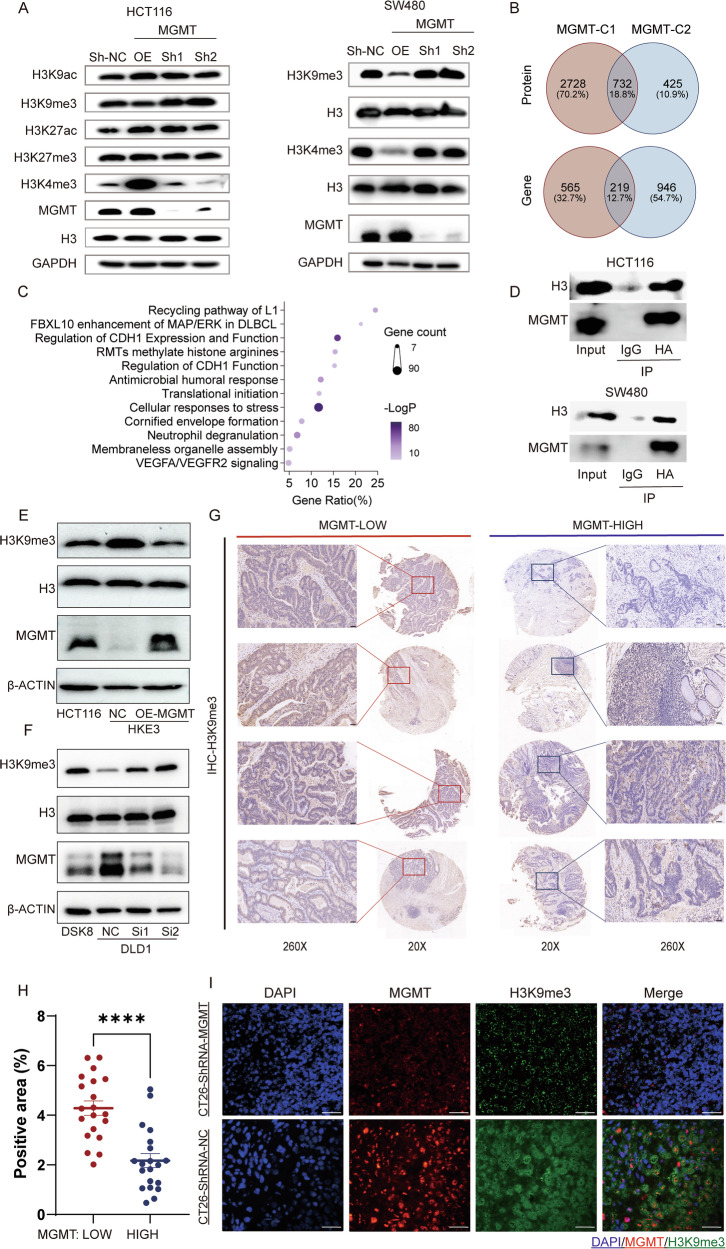
MGMT interacts with histone H3 and downregulates H3K9me3. **A** The levels of H3K9me3, H3K9ac, H3K27me3, H3K27ac, and H3K4me3 were determined by immunoblotting in HCT116 and SW480 cells with SiRNA-NC, SiRNA-MGMT, and overexpression MGMT plasmids. **B** 732 common proteins and 219 common genes were identified by two independent Co-IP with the antibody to HA-MGMT, followed by LC-MS/MS. **C** Pathway enrichment analysis of proteins interacting with MGMT identified by LC-MS/MS. **D** Co-IP with antibody to HA-MGMT, followed by SDS-PAGE and immunoblotting, showing endogenous H3 and MGMT. **E** Immunoblotting of H3K9me3 and MGMT in HCT116, HKE3, and HKE3-OE-MGMT. **F** Immunoblotting of H3K9me3 and MGMT in DLD1 and DSK8 cells treated with SiRNA-NC and SiRNA-MGMT. **G** The correlation of H3K9me3 level and MGMT expression was evaluated by IHC in CRC tissue microarray (*n* = 40, Scale bar: 50 μm). **H** Quantification of positive area of H3K9me3. **I** Immunofluorescence microscopy showing the co-localization of MGMT and H3K9me3 in tumor tissue from mice bearing subcutaneous CT26/CT26-ShRNA-MGMT (Scale bar: 100 μm). Error bar: mean ± SEM; ****P < 0.0001, determined by Kruskal–Wallis test.

Then we explore the mechanism of MGMT in KRAS mutant CRC metastasis. Given that the protein levels of E-cadherin, N-cadherin, and Vimentin were affected by MGMT (Figs. 1, 2), we hypothesized that MGMT may regulate EMT-inducing transcription factors. Previous studies have shown that MGMT catalyzes the transfer of methyl groups from O (6)-alkylguanine and other methylated moieties of the DNA to its own molecule, which repairs the toxic lesions [24]. Thus, we explored chromatin-based mechanisms. Firstly, we tested whether genome-wide methylation levels change when MGMT is regulated in CRC cells. However, the results showed that the global 5mC level did not change significantly when MGMT was overexpressed or knocked down in SW480 and HCT116 cells (Fig. S4A). Furthermore, we profiled multiple H3 modifications (H3K9me3, H3K9ac, H3K27ac, H3K27me3, H3K4me3) in SW480 and HCT116 CRC cells. The level of H3K9me3 decreased with MGMT overexpression and increased with MGMT knockdown in both HCT116 and SW480 (Fig. 5A). Notably, the Co-IP assays determined that MGMT interacts with histone H3 (Supplementary data 1). LC-MS/MS analysis shows that 732 common proteins (219 genes) were detected to interact with MGMT in two independent Co-IP experiments for HA-MGMT-HCT116 (Fig. 5B). KEGG analysis of 219 common genes suggested that the pathway *RMTs methylate histone arginine* was enriched, and Co-IP following western blot confirmed an interaction between MGMT and histone H3 (Fig. 5C, D).

Meanwhile, *Regulation of CDH1 Expression and Function* was enriched, further validating our finding that MGMT plays a role in regulating EMT (Fig. 5C). Moreover, DSK8 and HKE3 showed higher levels of H3K9me3 than their KRAS-mutant counterparts (DLD1 and HCT116). Consistently, increased levels of H3K9me3 were observed in DLD1 with knockdown of MGMT, while decreased levels of H3K9me3 were found in HKE3 with MGMT overexpression (Fig. 5E, F).

Our results further demonstrate that modulation of MGMT expression influences global H3K4me3 levels in a cell context-dependent manner. Notably, the observed effect differed between HCT116 and SW480 cells, suggesting that the genetic background may underlie the differential response (Fig. 5A). For instance, HCT116 cells carry wild-type p53, whereas SW480 cells harbor mutant p53, a factor known to influence chromatin modifier activity and epigenetic landscapes [25] significantly. This cell-specific variability underscores that the role of MGMT in regulating histone modifications is highly dependent on the cellular genetic context, consistent with our earlier findings regarding its non-canonical functions.

To further validate the correlation between MGMT and H3K9me3, we established a tissue microarray comprising 40 CRC patients (Table S7, Fig. S4B). Immunohistochemistry and transcriptomic data showed that H3K9me3 levels were negatively correlated with MGMT expression in CRC (Fig. 5G, H). We established orthotopic xenograft models by injecting MGMT-knockdown CT26 cells. Immunofluorescence analysis revealed increased levels of H3K9me3 in orthotopic cancer with MGMT knockdown (Fig. 5I). Collectively, these findings reveal a novel non-canonical epigenetic regulatory function of MGMT, which interacts with histone H3 and downregulates H3K9me3 in KRAS-mutant CRC.

### MGMT-mediated reduction of H3K9me3 promotes TWIST1 transcription

**Fig. 6:**
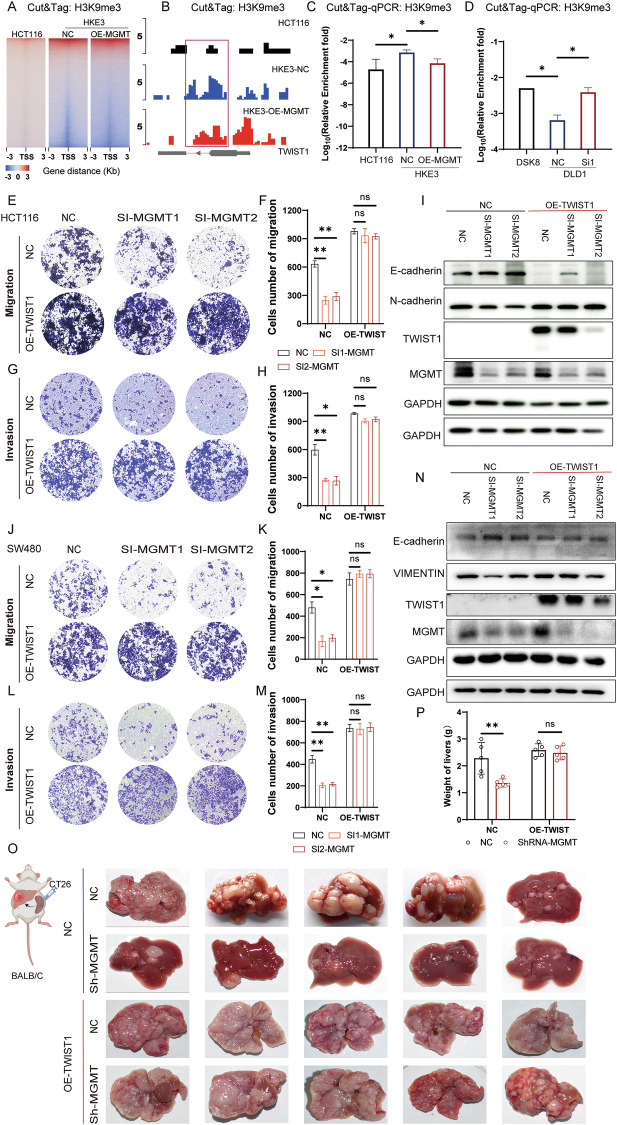
MGMT-mediated reduction of H3K9me3 promotes TWIST1 transcription. **A** Heatmap comparison of H3K9me3 signal over 3-kb windows centered around transcriptional start sites in HCT116, HKE3, and HKE3-OE-MGMT. **B** IGV tracks for TWIST1 from H3K9me3 CUT&Tag-seq analysis. **C** H3K9me3 levels at the TWIST1 promoter region by CUT&Tag-qPCR assay in HCT116, HKE3, and HKE3-OE-MGMT. **D** H3K9me3 levels at the TWIST1 promoter region by CUT&Tag-qPCR assays in DLD1 and DSK8 cells treated with SiRNA-NC and SiRNA-MGMT. **E–H** Transwell migration and Matrigel invasion assays showed that TWIST1 overexpression rescued the impaired invasion and migration caused by MGMT knockdown in HCT116. Quantification of migrated/invaded cells (*n* = 3 independent experiments). **I** Restored EMT markers expressions (E-cadherin, N-cadherin) were determined by immunoblotting in HCT116 and OE-TWIST1-HCT116. **J–M** Transwell migration and Matrigel invasion assays showed that TWIST1 overexpression rescued the impaired invasion and migration caused by MGMT knockdown in SW480. Quantification of migrated/invaded cells (*n* = 3 independent experiments). **N** Restored EMT markers expression (E-cadherin, Vimentin) was determined by immunoblotting in SW480. **O** TWIST1 overexpression abrogated the reduction in liver metastasis observed with MGMT knockdown in liver metastasis models by spleen injection. **P** Quantification of the weight of mouse livers from different groups. Values are presented as mean ± SEM. *P < 0.05, **p < 0.01, ns: non-significant, determined by one-way ANOVA (**C**, **D**, **F**, **M**, **K**, **H**) and two-tailed Welch’s *t* test (**P**).

To further investigate the regulatory role of H3K9me3, which is regulated by MGMT in gene transcription, we conducted CUT&Tag followed by sequencing (CUT&Tag-seq) with antibodies against H3K9me3 in HCT116, HKE3, and HKE3-OE-MGMT. Analysis results revealed that H3K9me3 were enriched in promoter regions (±3 kb around transcriptional start sites) for HKE3 compared with HCT116, and the abundance of H3K9me3 in promoter regions were decreased when MGMT was overexpressed in HKE3 (Fig. 6A). Given our previous findings that increased MGMT promote KRAS mutant CRC metastasis and downregulate H3K9me3, we hypothesized that MGMT promote EMT and regulate the expression of related genes expression in KRAS mutant CRC by downregulating H3K9me3. Hence, we analyzed the level of H3K9me3 at EMT-related gene promoters (Fig. 6B, Fig. S5A). The results showed increased H3K9me3 levels at the TWIST1 promoter region in HKE3 compared to HCT116 (Fig. 6B, Fig. S5B). Overexpressing MGMT in HKE3 significantly decreased H3K9me3 levels at the TWIST1 promoter region (Fig. 6B). These findings suggested that the increased MGMT in KRAS mutant CRC promoted TWIST1 transcription by regulating H3K9me3 levels in its promoter. Furthermore, we validated the results both in HCT116, HKE3, HKE3-OE-MGMT and DSK8, DLD1, DLD1-Si-MGMT by CUT&Tag-qPCR (Fig. 6C, D).

To further assess the role of TWIST1 in MGMT-mediated EMT for KRAS mutant CRC, we performed rescue experiments. Functionally, TWIST1 overexpression rescued the impaired invasion and migration caused by MGMT knockdown in HCT116 and SW480 (Fig. 6E–H, J–M) and restored EMT marker expression (E-cadherin, N-cadherin, Vimentin) in multiple cell lines (Fig. 6I, N). Consistently, liver metastatic mouse models (spleen injection of CT26-NC, CT26-ShMGMT, OE-TWIST1, OE-TWIST1-ShMGMT cells) revealed that TWIST1 overexpression abrogated the reduction in liver metastasis observed with MGMT knockdown. And there were no significant differences in liver metastatic lesions and liver weights between the MGMT knockdown groups and the control groups when TWIST1 was overexpressed in CT26 (Fig. 6O, P). These findings indicate that increased MGMT promotes migration, invasion, and EMT by upregulating TWIST1 in KRAS-mutant CRC. Collectively, these data indicate that MGMT promotes EMT and metastasis by decreasing H3K9me3 at the TWIST1 promoter and thereby enhancing TWIST1 transcription.

### MGMT is a potential therapeutic target to inhibit KRAS mutant CRC in combination with anti-EGFR drugs

**Fig. 7:**
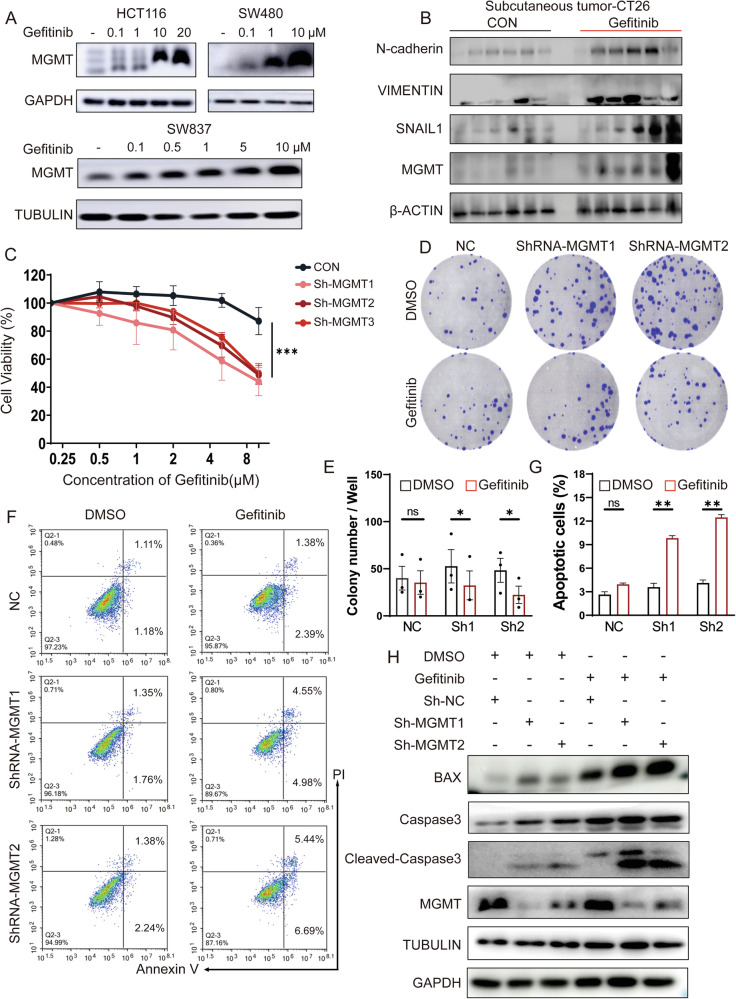
Targeting MGMT sensitizes KRAS-mutant CRC to anti-EGFR therapy. **A** Immunoblotting showed the level of MGMT protein in KRAS-mutant cell lines (HCT116, SW480, SW837) after Gefitinib treatment. **B** Immunoblotting of EMT-related proteins (N-cadherin, Vimentin, Snail1) and MGMT in subcutaneous CT26 tumors from mice treated with PBS (CON) / Gefitinib. **C** CCK-8 assay determined the IC50 values of Gefitinib for HCT116 and ShRNA-MGMT-HCT116. Tumor growth of HCT116 and ShRNA-MGMT-HCT116 treated by Gefitinib evaluated by (**D**) colony formation assay and (**E**) statistical analysis. **F**, **G** Annexin V/PI flow cytometry showed apoptosis cells in HCT116 and MGMT-knockdown cells following Gefitinib treatment. Quantification of apoptotic cells (*n* = 3 independent experiments). **H** Apoptosis-related proteins were determined by immunoblotting in HCT116 and ShRNA-MGMT-HCT116 treated with Gefitinib. Values are presented as mean ± SEM. *P < 0.05, **p < 0.01,***p < 0.001, ns: non-significant, determined by one-way ANOVA (**C**) and two-tailed Welch’s *t* test (**E**, **G**).

Given that MGMT is involved in EMT and that EMT is the leading cause of resistance to EGFR inhibitors, we evaluated the therapeutic potential of targeting MGMT in KRAS-mutant CRC. Firstly, RNA-seq analysis showed that MGMT expression was significantly increased and positively correlated with the EMT score in Gefitinib-treated cells (Fig. S6A, B). Western Blot showed MGMT protein increased in a dose-dependent manner after Gefitinib treatment in KRAS-mutant cell lines (HCT116, SW480, SW837 (mutant KRAS G12C), Fig. 7A). The tumor had increased MGMT expression from the CT26 subcutaneous tumor mice that were treated with Gefitinib compared with controls, along with a change in EMT markers expression (Fig. 7B). These findings indicate that MGMT induced EMT may contribute to anti-EGFR resistance in KRAS-mutant CRC.

To identify the MGMT responsible for anti-EGFR resistance in KRAS-mutant CRC, cell viability and colony formation assays were performed. MGMT knockdown sensitized HCT116 and SW480 cells to Gefitinib, as evidenced by decreased IC50 and less colony formation (Fig. 7C–E, Fig. S6C). We then used Annexin V/PI flow cytometry to characterize apoptosis in *MGMT* knockdown cells following Gefitinib treatment. The results showed a significant increase in apoptotic cells in MGMT-knockdown cells following Gefitinib treatment compared with controls (Fig. 7F, G, Fig. S6D-E). Consistently, Cleaved Caspase-3 and BAX showed a marked increase in MGMT knockdown cells treated with Gefitinib (Fig. 7H, Fig. S6F).

**Fig. 8:**
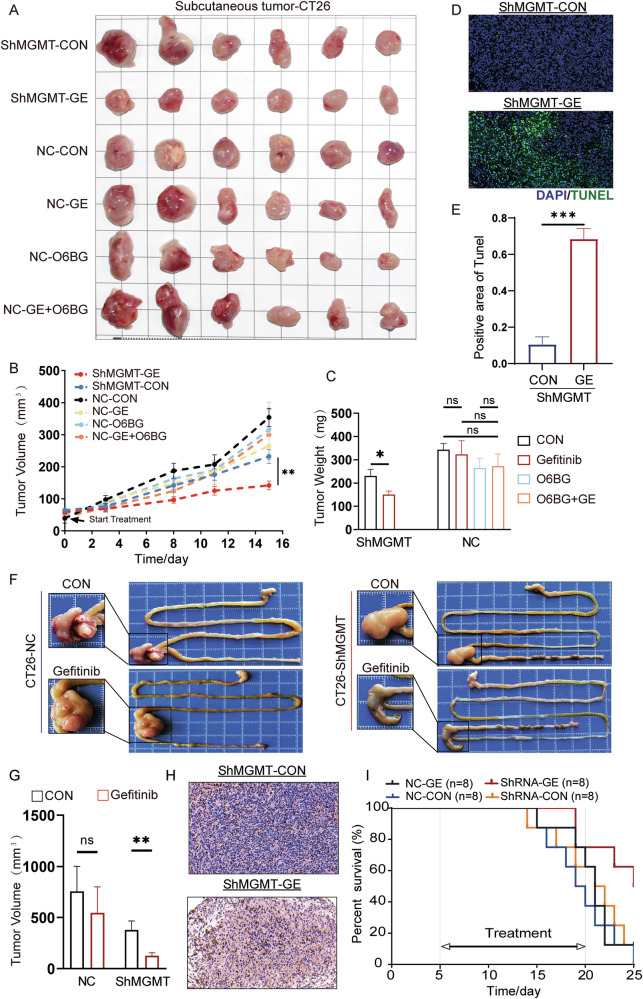
MGMT is a potential therapeutic target to inhibit KRAS mutant CRC in combination with anti-EGFR drugs. **A** Representative images of CT26 and ShRNA-MGMT-CT26 xenografts from mice treated with PBS/Gefitinib/O6-Benzylguanine (*n* = 6 mice per group). MGMT knockdown plus Gefitinib suppressed the growth of CT26 xenografts, as evidenced by significant reductions in tumor volume (**B**) and weight (**C**). **D** IF staining of TUNEL compared cell proliferation in ShRNA-MGMT-CT26 xenografts treated by PBS or Gefitinib (Scale bar: 20 μm). **E** Quantification of positive area of TUNEL. **F** Survival curve showed the time to reach 2000 mm^3^ tumor volume in mice bearing ShRNA-NC/ShRNA-MGMT-CT26 xenografts treated with Gefitinib or PBS. ShRNA-MGMT plus Gefitinib more significantly suppressed CT26 orthotopic tumors (**G**), as evidenced by significant reductions in tumor volume (**H**) and lower Ki-67 expression determined by IHC staining (**I**; Scale bar: 20 μm). Values are presented as mean ± SEM. *P < 0.05, **p < 0.01,***p < 0.001, ns: non-significant, determined by one-way ANOVA (**B**, **C**), two-tailed Welch’s *t* test (**E**, **H**), and Log-rank test (**F**).

We constructed subcutaneous tumors and cecum orthotopic tumors in mouse models to further investigate the therapeutic effects of targeting MGMT in combination with anti-EGFR drugs in KRAS mutant CRC. The mice bearing subcutaneous CT26 tumors were treated with Gefitinib or O6-Benzylguanine or combination therapy and for 15 days. However, there were no differences in tumor growth rate or tumor volume between the single-agent groups, the combination therapy groups, and the controls (Fig. 8A–C). Furthermore, we observed that O6-Benzylguanine treatment had no effect on MGMT or EMT marker expression (Fig. S6G). These data demonstrate that MGMT regulates EMT in KRAS-mutant cells by interacting with H3 histone, a function distinct from its traditional methyltransferase activity reported in current studies and highly consistent with our previous results.

In addition, mice bearing subcutaneous MGMT-knockdown CT26 tumors showed significant suppression with Gefitinib treatment. Our results showed that both subcutaneous and orthotopic tumors derived from MGMT-knockdown CT26 cells responded with reduced tumor volume and weight (Fig. 8A–C, F, G, Fig. S7). Notably, Immunofluorescence and IHC analysis of the tumor samples revealed that Gefitinib significantly increased apoptotic cells and decreased the proportion of Ki-67-positive cells in MGMT-knockdown tumors (Fig. 8D, E, H). A more pronounced Gefitinib therapeutic effect was observed in the survival curve, as evidenced by the longer time required to reach 2000 mm^3^ tumor volume in the MGMT-knockdown group compared to the control group (Fig. 8I). These data suggest that abundance of MGMT, rather than its canonical enzymatic activity alone, contributes to anti-EGFR resistance. Reducing MGMT levels augments the efficacy of EGFR inhibitors.

## Discussion

We report that MGMT is upregulated in KRAS-mutant CRC and drives metastasis by promoting EMT through an epigenetic mechanism. This study revealed a novel biological function of MGMT, which interacts with histone H3 and reduces H3K9me3 at the TWIST1 promoter, increasing TWIST1 transcription and activating EMT. Our orthotopic liver metastasis model recapitulates clinically relevant metastatic behavior and supports a mechanistic link between KRAS mutation, MGMT upregulation, and metastatic progression.

KRAS is one of the most frequently mutated oncogenes in metastatic CRC, and its mutation testing is a routine clinical practice before treating metastatic cases [25]. Patients with KRAS-mutant CRC have a later stage and poorer prognosis than those with KRAS wild-type CRC [26]. The mutant KRAS gene results in constitutive activation of the KRAS protein, which acts as a molecular switch, persistently stimulating downstream signaling pathways in cancer cells. Our findings, for the first time, showed that KRAS-mutant CRC exhibited increased MGMT expression, which was associated with liver metastasis. This presents important perspectives on the role of increased MGMT in KRAS-mutant CRC, a novel epigenetic modification that promotes the expression of TWIST1 by decreasing H3K9me3 levels at its promoter region. Furthermore, reducing MGMT levels in cells effectively suppressed KRAS-mutant CRC proliferation and metastasis.

The regulation of histone modification is essential for a number of nuclear processes, including transcription, replication, and repair, and epigenetic regulators play critical roles in tumor initiation, progression, and therapy in KRAS mutant cancer [27]. For example, histone lysine demethylase PHF8 up-regulated the expression of PD-L1, KRAS, BRAF, and c-MYC by increasing the levels of transcriptional activation marks H3K4me3 and H3K27ac within their promoter regions. Targeting PHF8 substantially improved the efficacy of anti-PD1 therapy and inhibited the malignant phenotypes of KRAS- or BRAF-mutant CRC cells [28]. H3K4me2/3 demethylases KDM5D were identified as a potential basis for sex-specific differences in KRAS-mutant CRC progression, and KRAS*-STAT4-KDM5D drives invasion and metastases by repressing genes governing cell-cell junction complex integrity and CD8^+^ T cell-mediated anti-tumor immunity [29]. Epigenetic regulation is central to KRAS-driven oncogenesis. Consistently, our study highlights an important role of epigenetic regulators in promoting KRAS-mutant CRC metastasis and reducing therapy resistance, and adds MGMT to this list. It is noteworthy that MGMT overexpression is associated with liver metastasis in CRC patients, and global 5mC did not change with MGMT perturbation, suggesting that the pro-metastatic role of MGMT here is distinct from its canonical DNA-repair activity. Co-IP followed by mass spectrometry (Co-IP/MS) confirmed the physical association between MGMT and histone H3. Functionally, our CUT&Tag data demonstrated that up-regulation of MGMT in KRAS-mutant CRC cells reduced global and locus-specific (e.g., the TWIST1 promoter) H3K9me3 levels, while MGMT knockdown increased them. This modulation of H3K9me3 consequently enhanced chromatin accessibility and facilitated the transcription of downstream genes such as TWIST1. Our study provides new insights into MGMT as a novel epigenetic checkpoint for H3K9me3, and MGMT downregulates H3K9me3 levels through a direct protein interaction with histone H3. However, the precise enzymatic mechanism remains unclear.

Furthermore, the discovery of MGMT’s epigenetic role in KRAS-mutant CRC invites discussion on its potential as a predictive biomarker for intrinsic or acquired resistance to EGFR inhibitors. Patients with KRAS-mutant CRC could not benefit from EGFR inhibitor therapy, which is used as a first-line regimen in patients with advanced or metastatic colorectal cancer [30]. Our data showed that MGMT is a potential therapeutic target to inhibit KRAS-mutant CRC in combination with anti-EGFR drugs, and that considerable therapeutic effects were observed in both subcutaneous and cecum orthotopic tumors in mouse models. Meanwhile, we found that the therapeutic effect does not depend on MGMT enzyme activity but rather on the concentration of MGMT protein in the cancer. Consequently, assessing MGMT levels in KRAS-mutant CRC patients might help stratify those with a higher likelihood of primary resistance to EGFR inhibitors or those at risk of developing acquired resistance during combination regimens. Future clinical studies validating MGMT protein or mRNA expression as a companion biomarker would be valuable in personalizing therapeutic approaches for KRAS-mutant CRC.

Finally, while our study delineates a direct interaction between MGMT and histone H3 leading to H3K9me3 reduction, whether MGMT acts as a cryptic histone demethylase, a scaffolding platform for epigenetic modifiers, or a competitive binder remains to be determined. Future structural and biochemical studies will be essential to unravel this non-canonical activity. Moreover, it may also be worth considering whether the role of MGMT varies across different genetic backgrounds, such as different p53 contexts. Investigating MGMT’s role within defined molecular subtypes of CRC will further refine its potential as a context-dependent therapeutic target.

## Supplementary information

Supplementary Figures

Supplementary Tables

Unprocessed WB

Dataset 1

Dataset 2

Dataset 3

Dataset 4

Dataset 5

## Data Availability

The RNA-seq and Cut&Tag data from cell lines generated in this study are available in the NCBI SRA database under accession PRJNA1405327. The RNA-seq data of CRC cohort 1 is available in the GSA for Human database under accession numbers HRA016519 and HRA016480. This paper does not report custom code. Any additional information required to reanalyze the data reported in this work is available from the lead contact upon request.
