# Supplementary material for: Methyltransferase MGMT upregulation drives metastasis by activating epithelial-mesenchymal transition in KRAS mutant colon cancer
Source: Cell Death Dis. 2026 May 16;17(1):628. doi: 10.1038/s41419-026-08858-z (PMC13346676; doi:10.1038/s41419-026-08858-z)
Supplement: Supplementary file 1 — Supplementary Figures [file 41419_2026_8858_MOESM1_ESM.docx]

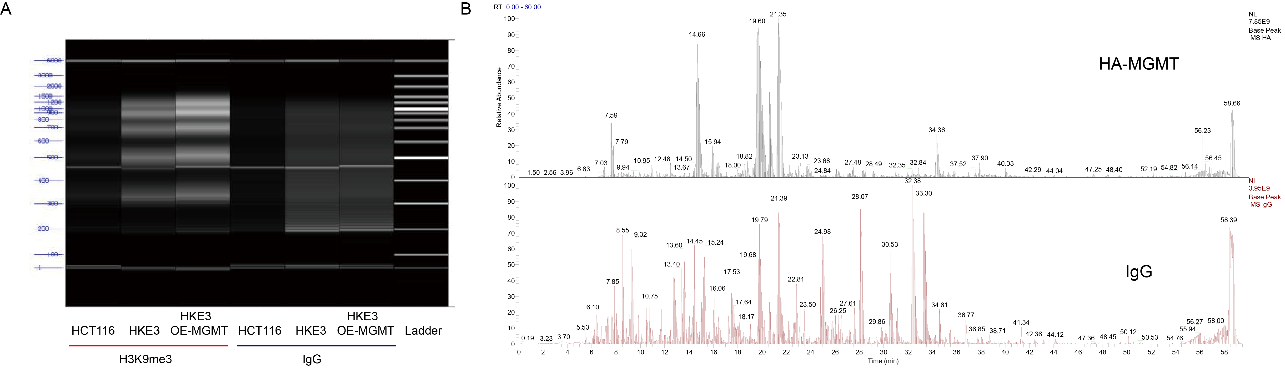


Fig. S1: A. Gel image for DNA from CUT&Tag to H3K9me^3^ and IgG. B. Base peak of Co-IP experiments for HA-MGMT-HCT116 and IgG.


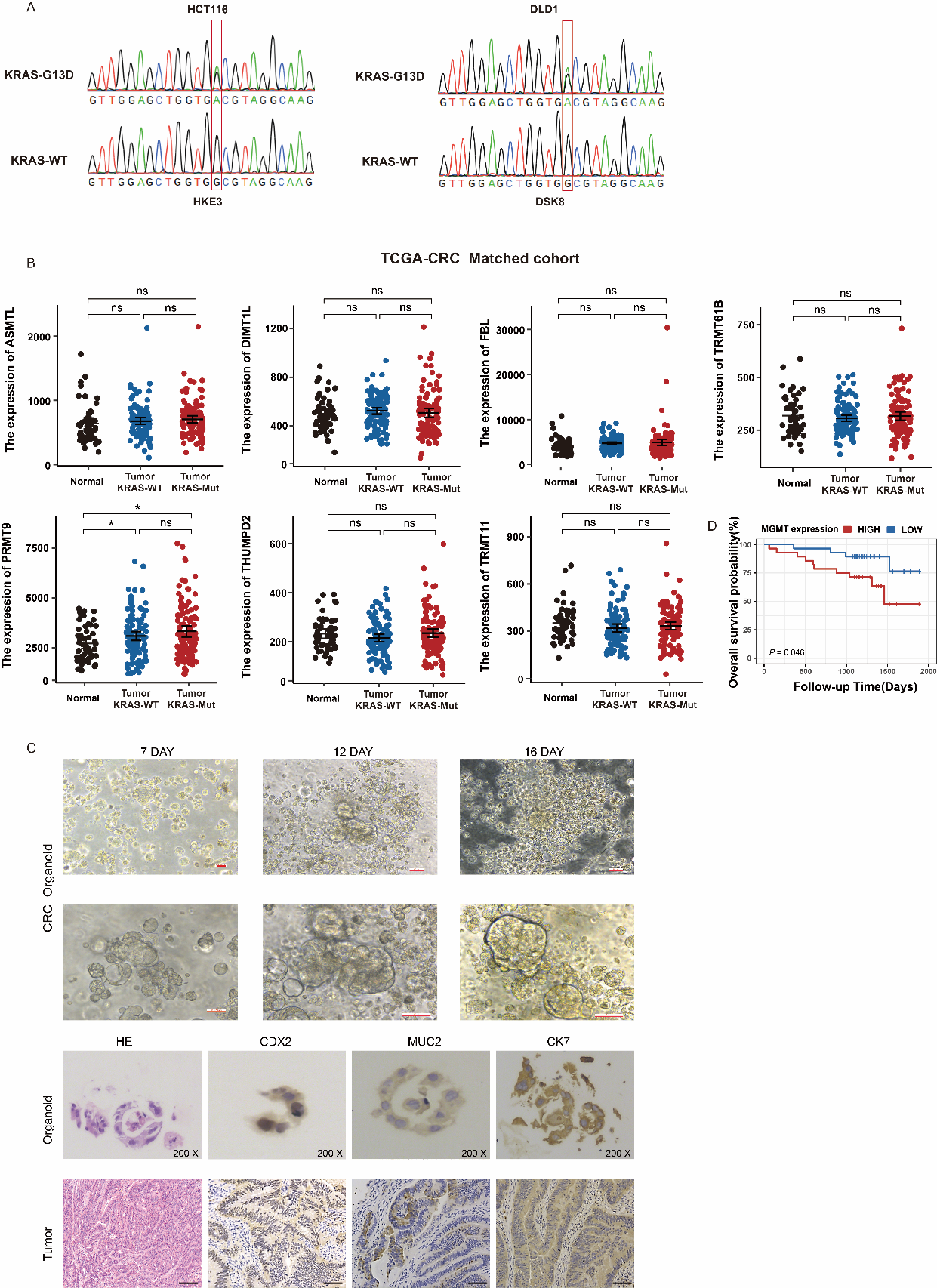


Fig. S2: A. The mutant sequence of KRAS in HCT116/HKE3 and DLD1/ DSK8. B. The expression of eight genes involved in the *methylation pathway* in gender- and age-matched TGCA cohort. C. Representative images, and HE stained CRC organoid; ICH for CDX2, MUC2, and CK7 were performed to confirm the colon cells. D. Kaplan-Meier survival curves contrasting the difference between KRAS-mutant CRC patients with low and high MGMT levels. Values are presented as mean ± SD. ** *P*< 0.01, determined by one-way ANOVA.


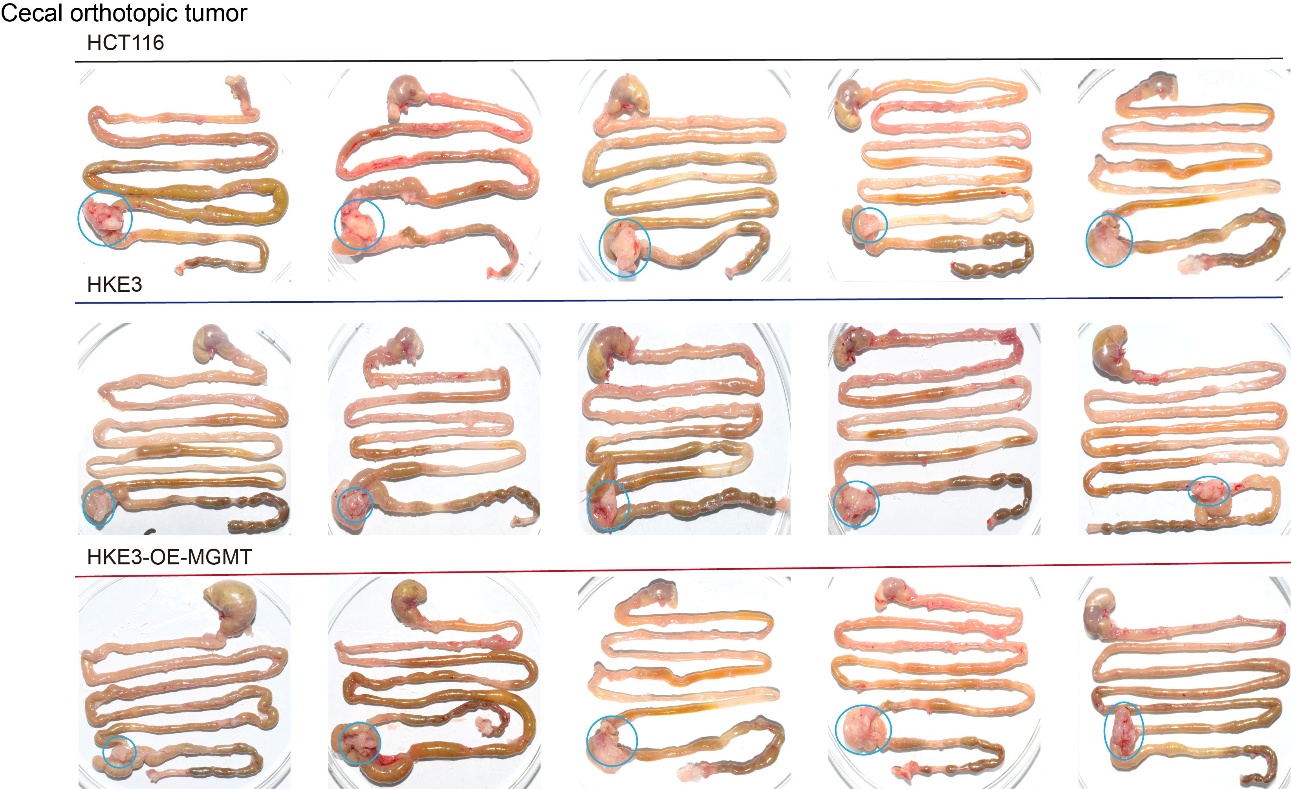
Fig. S3: Representative images of cecal orthotopic tumor from orthotopic CRC metastatic model mice injected with HCT116, HKE3, and HKE3-OE-MGMT cells (n = 5 per group).


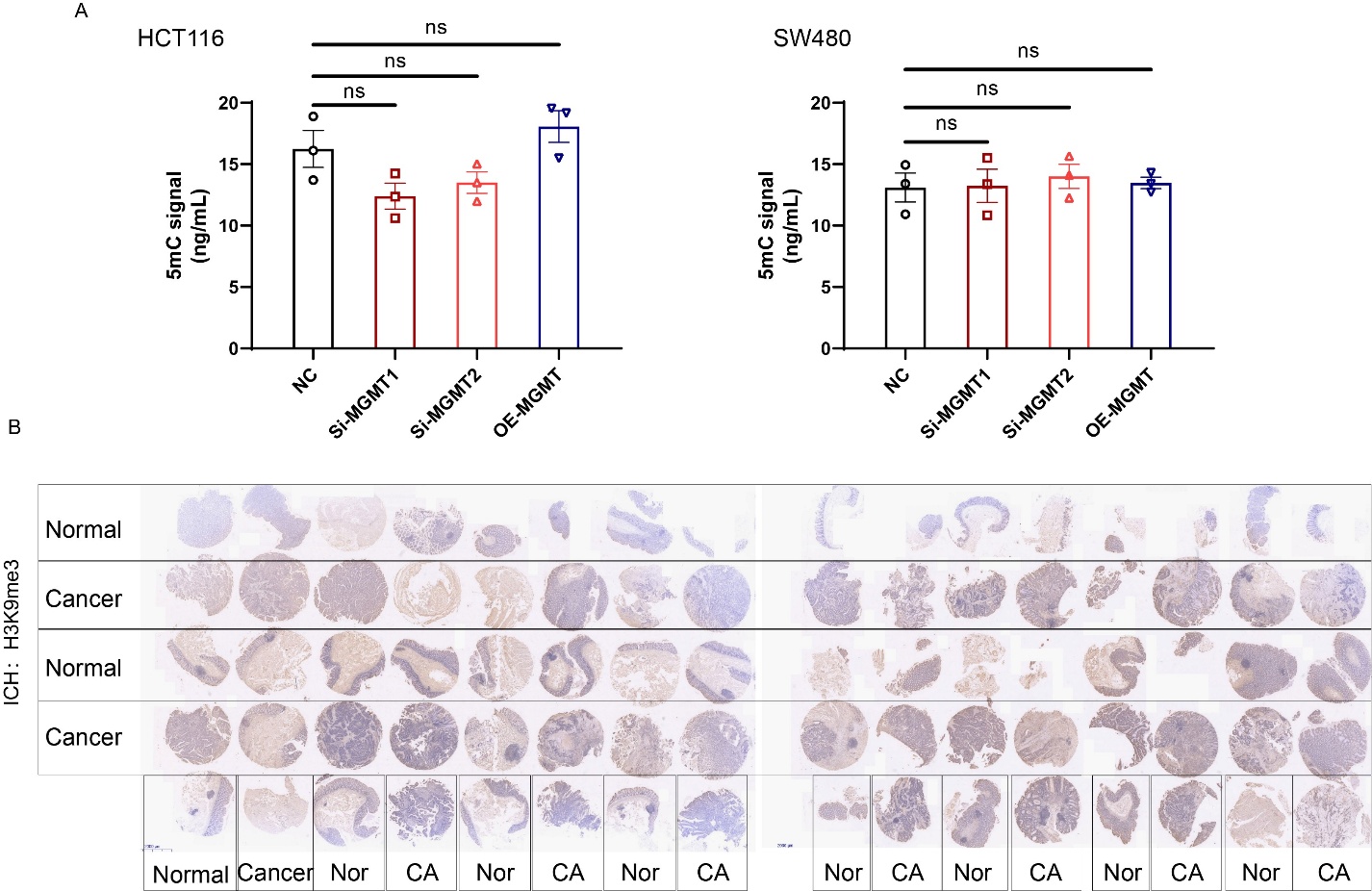


Fig. S4: A. The global 5mC level in HCT116 and SW480 treated with Si-MGMT and OE-MGMT. B. The correlation between H3K9me3 levels and MGMT expression was evaluated by IHC in CRC tissue microarray (n=40). Values are presented as mean ± SEM. ns: non-significant, determined by one-way ANOVA.


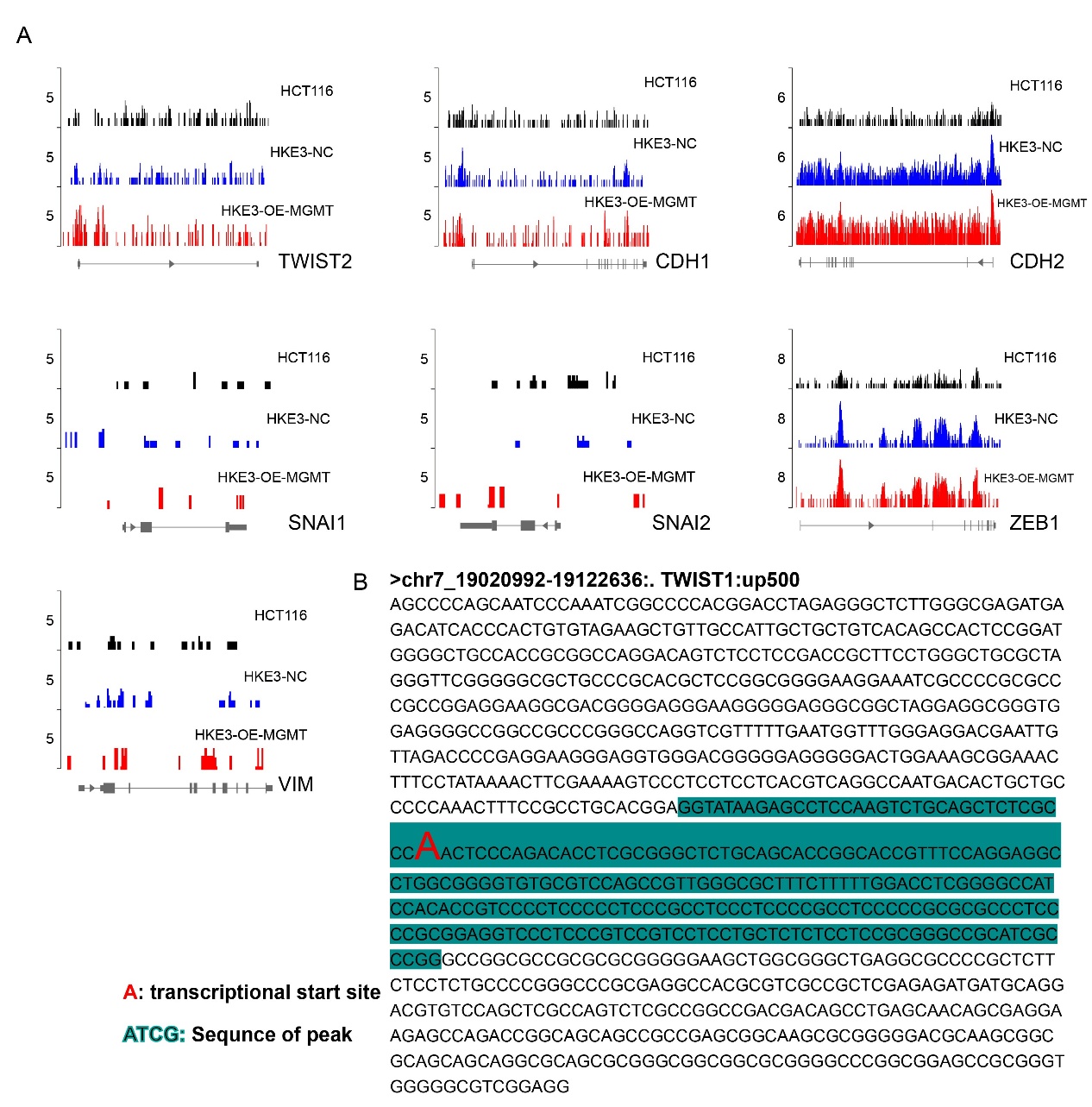


Fig. S5: A. IGV tracks for EMT and regulates related genes from H3K9me3 CUT&Tag-seq analysis. B. H3K9me3 binding sequence in the promoter of TWIST1.


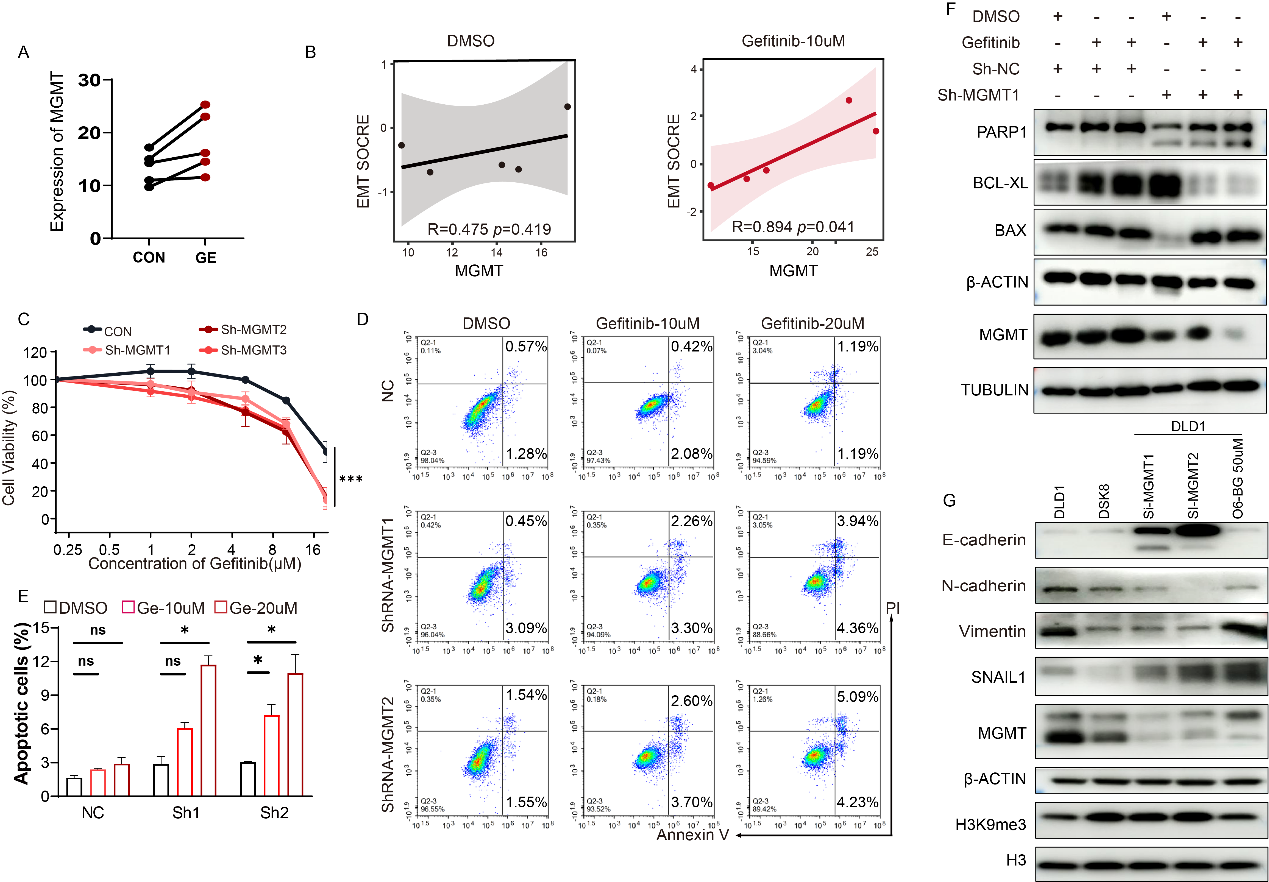


Fig. S6. Targeting MGMT sensitizes KRAS-mutant CRC to anti-EGFR therapy. A. The expression of MGMT increased after gefitinib treatment in KRAS-mutant cell lines (HCT116, SW837). B. The correlation of EMT score and MGMT expression was positive after gefitinib treatment in KRAS-mutant cell lines (HCT116, SW837). C. MGMT knockdown reduced IC50 values for gefitinib in SW480, which were determined by CCK8 assay. D-E. Annexin V/PI flow cytometry showed increased apoptosis in MGMT-knockdown SW480 cells following gefitinib treatment. Quantification of apoptotic cells. F. Increases in cleaved PARP1 and BAX in ShRNA-MGMT-SW480 treated with Gefitinib were determined by Immunoblotting. G. Immunoblotting of epithelial-mesenchymal transition-related proteins (N-cadherin, E-cadherin, Vimentin, Snail) and the level of H3K9me3 in DSK8 and DLD1 cells treated with siNC, si-MGMT, and O6-BG. Values are presented as mean ± SEM. **P* < 0.05, ****p* < 0.001, ns: non-significant, determined by one-way ANOVA (C) and two-tailed Welch’s t-test (E).


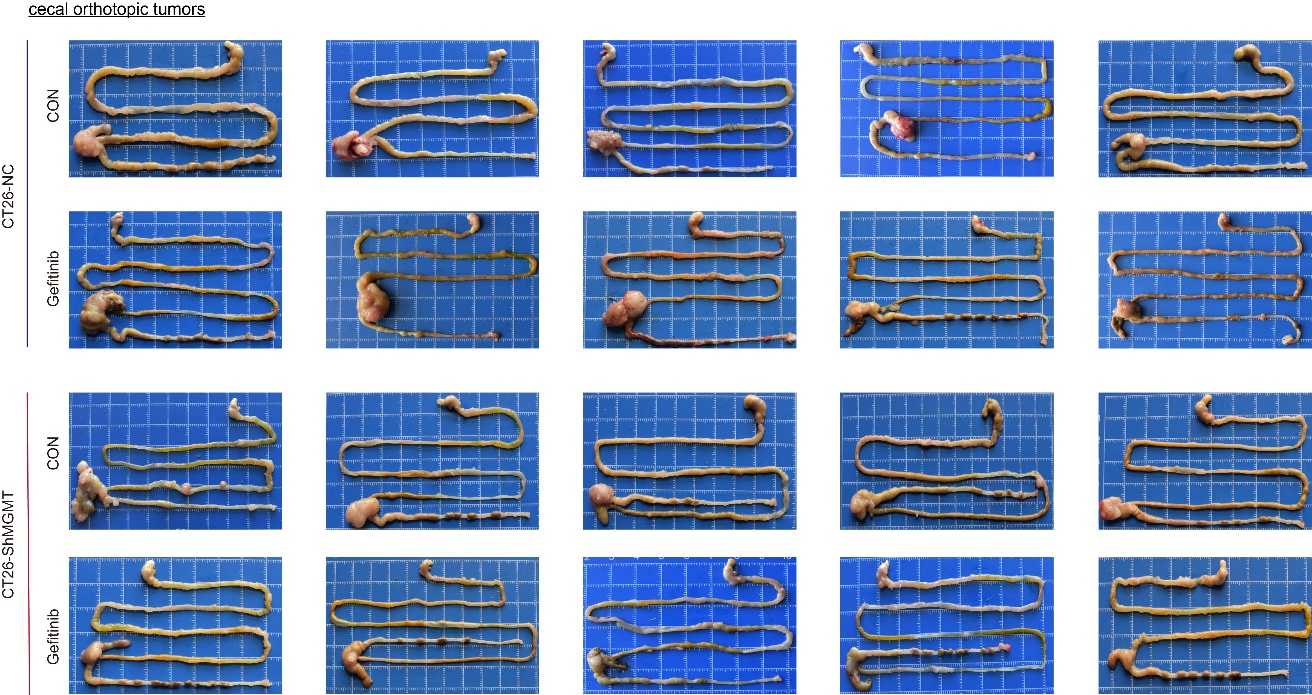


Fig. S7. Representative images of CT26/ShMGMT-CT26 cecal orthotopic tumors from mice treated with PBS and Gefitinib (n = 5 per group).
