# Supplementary material for: Methyltransferase MGMT upregulation drives metastasis by activating epithelial-mesenchymal transition in KRAS mutant colon cancer
Source: Cell Death Dis. 2026 May 16;17(1):628. doi: 10.1038/s41419-026-08858-z (PMC13346676; doi:10.1038/s41419-026-08858-z)
Supplement: Supplementary file 2 — Supplementary Tables [file 41419_2026_8858_MOESM2_ESM.docx]

Table S1. List of sgRNA, shRNA, and siRNA sequences used

| **Gene** | **siRNA or ShRNA target sequence** |
| --- | --- |
| h_MGMT_Si1 | AAAUAAAGCUCCUGGGCAATT |
| m_*h_MGMT_Si2* | UGCUGAAGGUUGUGAAAUUTT |
| m_h_MGMT-shRNA1 | CAAGGATTGTGAAATGAAA |
| h_MGMT-shRNA2 | GACAAGGATTGTGAAATGA |
| m_h_MGMT-shRNA3 | GTTCACCAGACAGGTGTTA |
| m_KRAS-sgRNA | GTGGTTGGAGCTGGTGGCGT |

Table S2. List of primers used for RT-PCR/qPCR assays

| **Classification** | **Primer (mouse)** | **Sequence (5' to 3')** |
| --- | --- | --- |
| GAPDH | h_GAPDH-F | TGGACCTGACCTGCCGTCTAGAAA |
|  | h_GAPDH-R | GTGGGTGTCGCTGTTGAAGTCAGA |
| MGMT | h_MGMT_F | CGTTTTCCAGCAAGAGTCGT |
|  | h_*MGMT*_R | ATGGGGACAGGATTGCCTCTC |
| KRAS | h_KRAS1-F | CGATACACGTCTGCAGTCAACT |
|  | h-KRAS1-R | CAAAGAATGGTCCTGCACCAG |
| CUT&Tag-qPCR | Spike-F | GCCTTCTTCCCATTTCTGATCC |
|  | Spike-R | CACGAATCAGCGGTAAAGGT |
|  | h_TWIST1-F6 | GCCCAACTCCCAGACACC |
|  | h_TWIST1-R6 | CCGAGGTCCAAAAAGAAAGCG |
|  | h_TWIST1-F8 | CCAAGTCTGCAGCTCTCGC |
|  | h_-TWIST1-R8 | TCCAAAAAGAAAGCGCCCAAC |
|  | h_TWIST1-F1 | CCAACTCCCAGACACCTCG |
|  | h_TWIST1-R1 | AAAAAGAAAGCGCCCAACGG |

Table S3. Antibodies used for Immunoblotting analysis

| Name | Sources | Identifier | Concentration |
| --- | --- | --- | --- |
| N-CA | Cell Signaling Technology | 13116S | 1:800 |
| E-CA | Cell Signaling Technology | 14472S | 1:1000 |
| Vimentin | Cell Signaling Technology | 5741S | 1:1000 |
| MGMT | Proteintech | 17195-1-AP | 1:800 |
| Snail1 | Cell Signaling Technology | 3879S | 1:1000 |
| Twist1 | Proteintech | 25465-1-AF | 1:1000 |
| Bax | Proteintech | 50599-2-Ig | 1:1000 |
| Caspase3 | Cell Signaling Technology | 9662S | 1:800 |
| Cleaved-Caspase3 | Cell Signaling Technology | 9661S | 1:800 |
| H3K9me3 | Cell Signaling Technology | 13969S | 1:1000 |
| H3K9ac | Cell Signaling Technology | 9649S | 1:1000 |
| H3K27me3 | Cell Signaling Technology | 9733S | 1:1000 |
| H3K27ac | Cell Signaling Technology | 8173S | 1:1000 |
| H3K4me3 | Cell Signaling Technology | 9751S | 1:1000 |
| Gapdh | Proteintech | 60004-1-AP | 1:10000 |
| β-actin | Proteintech | 66009-1-AP | 1:10000 |
| Histone 3 | Proteintech | 17168-1-AP | 1:10000 |
| HRP-conjugated Goat Anti-Rabbit IgG(H+L) | Proteintech | SA00001-2 | 1:10000 |
| HRP-conjugated Goat Anti-Mouse IgG(H+L) | Proteintech | SA00001-1 | 1:10000 |

Table S4. The clinical characteristics of the study population from TCGA

|  | **Normal** | **Tumor** | | ***P* Value** |
| --- | --- | --- | --- | --- |
|  |  | **KRAS-WT** | **KRAS-Mut** |  |
| No. of patient | 51 | 102 | 102 |  |
| Age |  |  |  | 0.969 |
| <70 | 22 | 46 | 45 |  |
| >70 | 29 | 56 | 57 |  |
| Gender |  |  |  | 0.469 |
| Female | 28 | 63 | 62 |  |
| Male | 23 | 39 | 40 |  |
| Primary site |  |  |  | 0.109 |
| Colon | 42 | 75 | 75 |  |
| Rectosigmoid junction | 7 | 16 | 11 |  |
| Rectum | 2 | 11 | 16 |  |
| pT |  |  |  | 0.414 |
| pT1 or pT2 | / | 19 | 14 |  |
| pT3 or pT4 | / | 83 | 88 |  |
| pN |  |  |  |  |
| pN0 | / | 59 | 58 | 0.993 |
| pN1 or pN2 | / | 43 | 44 |  |
| Tumor stage |  |  |  | 0.993 |
| **Ⅰ** or **Ⅱ** | / | 59 | 58 |  |
| **Ⅲ** or **Ⅳ** | / | 43 | 44 |  |

Table S5. Clinic characteristics of 56 CRC cohort for survival analysis

|  | **CRC(n=56)** | | ***P* Value** |
| --- | --- | --- | --- |
|  | **MGMT-Low** | **MGMT-High** |  |
| No. of patient | 28 | 28 |  |
| Age |  |  | 0.554 |
| <70 | 20 (71.4%) | 22(78.6%) |  |
| >70 | 8(28.6%) | 6 (21.4%) |  |
| Gender |  |  | 0.596 |
| Female | 16(57.1%) | 14(50.0%) |  |
| Male | 12(42.9%) | 14(50.0%) |  |
| Primary site |  |  | 0.582 |
| Colon | 18 (64.3%) | 16 (57.1%) |  |
| Rectum | 10(35.7%) | 12 (42.9%) |  |
| pT |  |  | 0.319 |
| pT1 or pT2 | 4(14.3%) | 7 (25.0%) |  |
| pT3 or pT4 | 24 (85.7%) | 21(75.0%) |  |
| pN |  |  | 0.027 |
| pN0 | 12(40.0%) | 4(14.3%) |  |
| pN1 or pN2 | 18(60.0%) | 24(85.7%) |  |
| Tumor stage |  |  | 0.027 |
| Ⅰ or Ⅱ | 12(40.0%) | 4(14.3%) |  |
| Ⅲ or Ⅳ | 18(60.0%) | 24(85.7%) |  |

Table S6. Baseline characteristics of 88 CRC cohort

|  | | **CRC(n=88)** | |
| --- | --- | --- | --- |
| Age | <70 | | ≥70 |
|  | 65(73.4%) | | 23 (26.6%) |
| Gender | Female | | Male |
|  | 40(45.5%) | | 48 (54.5%) |
| Primary site | Colon | | Rectum |
|  | 53(60.2%) | | 35(39.8%) |
| pT | pT1 or pT2 | | pT3 or pT4 |
|  | 13(14.8%) | | 75(85.2%) |
| pN | pN0 | | pN1 or pN2 |
|  | 49(55.7%) | | 39(44.3%) |
| Tumor stage | **Ⅰ** or **Ⅱ** | | **Ⅲ** or **Ⅳ** |
|  | 49(55.7%) | | 39(44.3%) |

Table S7. The clinical characteristics of the study population for CRC tissue microarray

|  | **CRC(n=40)** | | ***P* Value** |
| --- | --- | --- | --- |
|  | **MGMT-Low** | **MGMT-High** |  |
| No. of patient | 20 | 20 |  |
| Age |  |  | 0.695 |
| <70 | 15(75.0%) | 13(65.0%) |  |
| >70 | 5(25.0%) | 7 (35.0%) |  |
| Gender |  |  | 0.474 |
| Female | 11(55.0%) | 8(40.0%) |  |
| Male | 9(45.0%) | 12(60.0%) |  |
| Primary site |  |  | 1.000 |
| Colon | 11 (55.0%) | 11 (55.0%) |  |
| Rectum | 9(45.0%) | 9 (45.0%) |  |
| pT |  |  | 0.605 |
| pT1 or pT2 | 5(25.0%) | 4 (20.0%) |  |
| pT3 or pT4 | 15 (75.0%) | 16 (80.0%) |  |
| pN |  |  | 0.279 |
| pN0 | 13(65.0%) | 9(45.0%) |  |
| pN1 or pN2 | 7(35.0%) | 11(55.0%) |  |
| Tumor stage |  |  | 0.279 |
| Ⅰ or Ⅱ | 13(65.0%) | 9(45.0%) |  |
| Ⅲ or Ⅳ | 7(35.0%) | 11(55.0%) |  |
