# Supplementary material for: Methyltransferase MGMT upregulation drives metastasis by activating epithelial-mesenchymal transition in KRAS mutant colon cancer
Source: Cell Death Dis. 2026 May 16;17(1):628. doi: 10.1038/s41419-026-08858-z (PMC13346676; doi:10.1038/s41419-026-08858-z)
Supplement: Supplementary file 3 — Unprocessed WB [file 41419_2026_8858_MOESM3_ESM.pptx]

## Slide 1
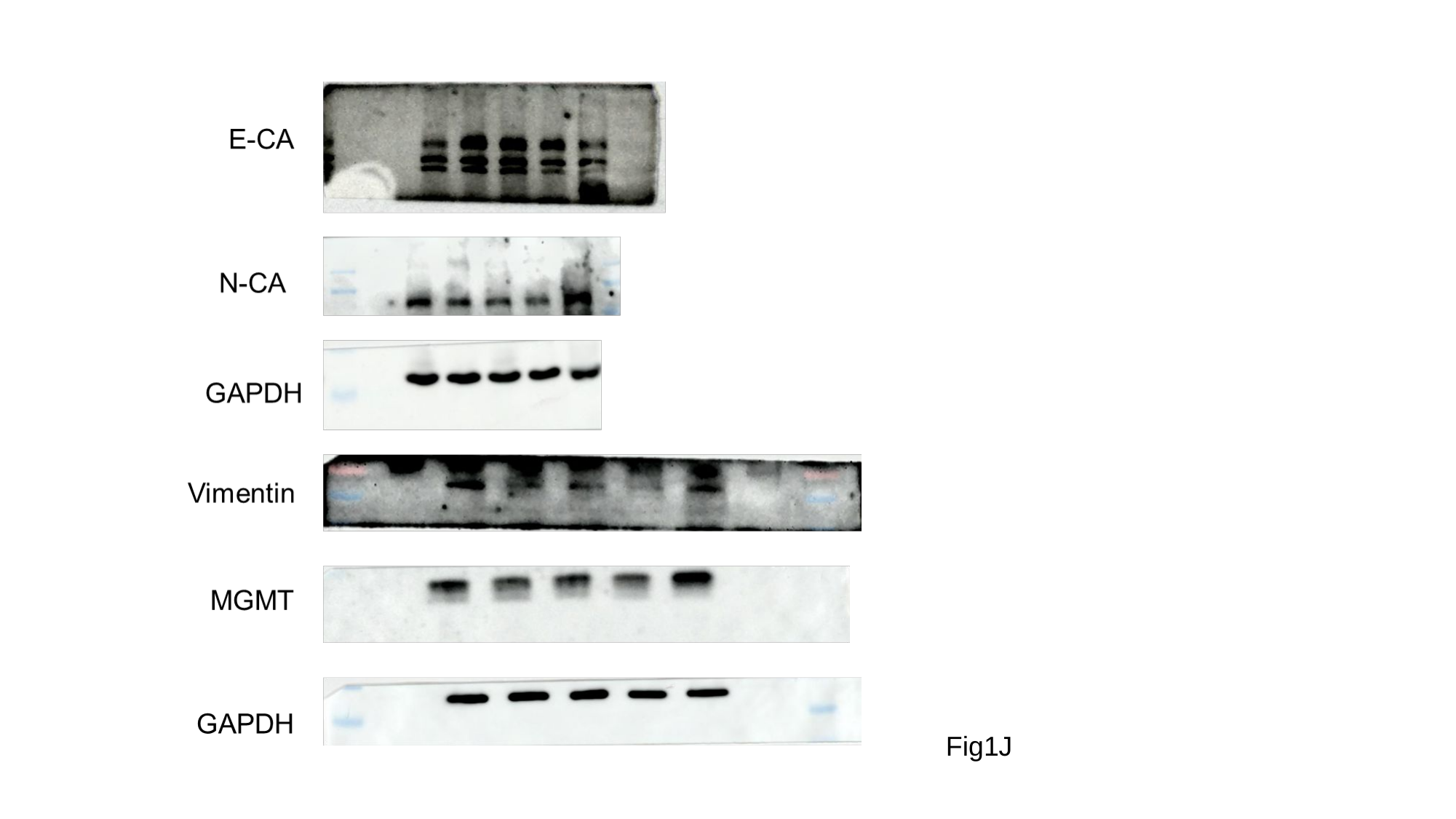

Fig1J

## Slide 2
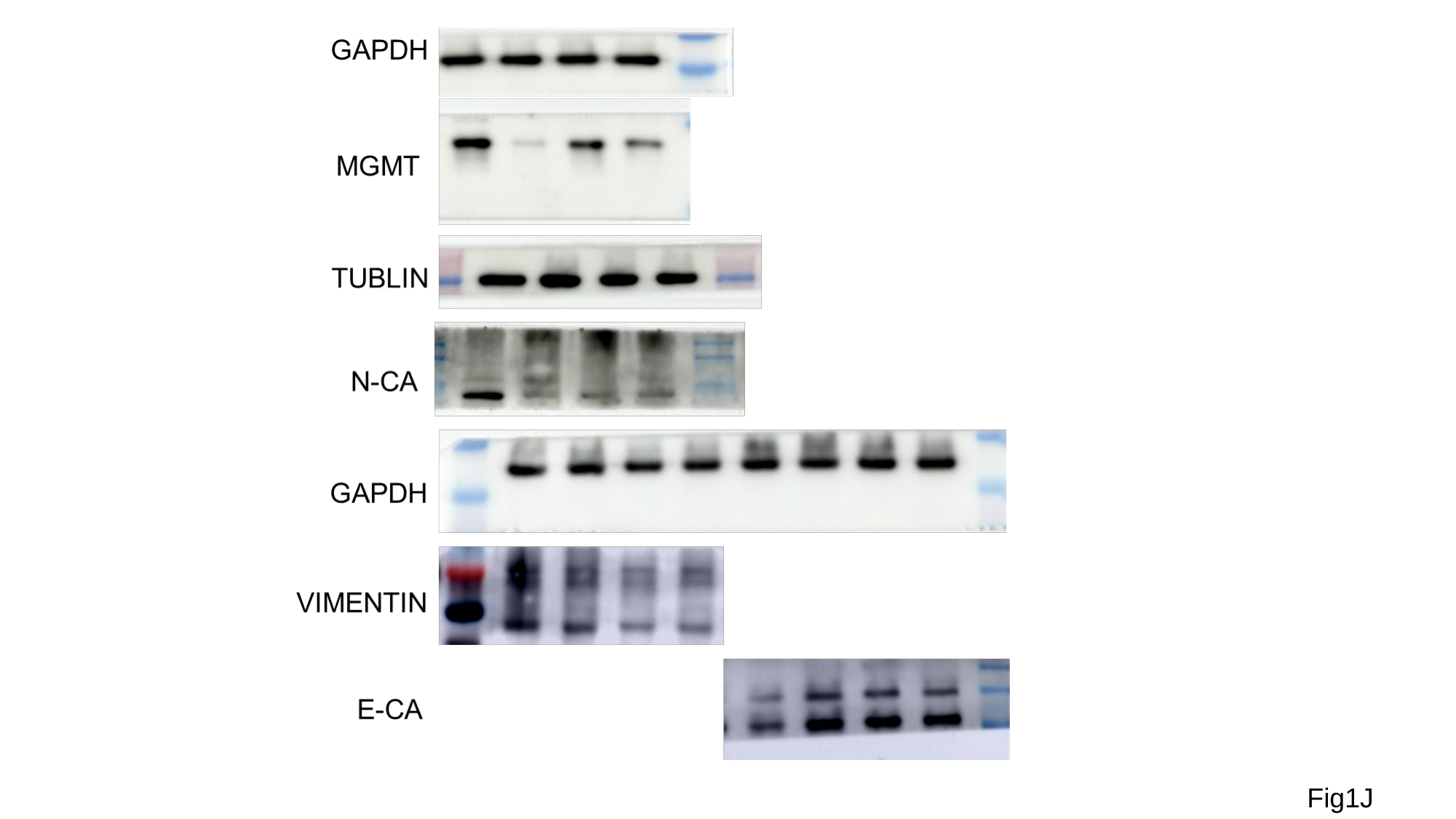

Fig1J

## Slide 3
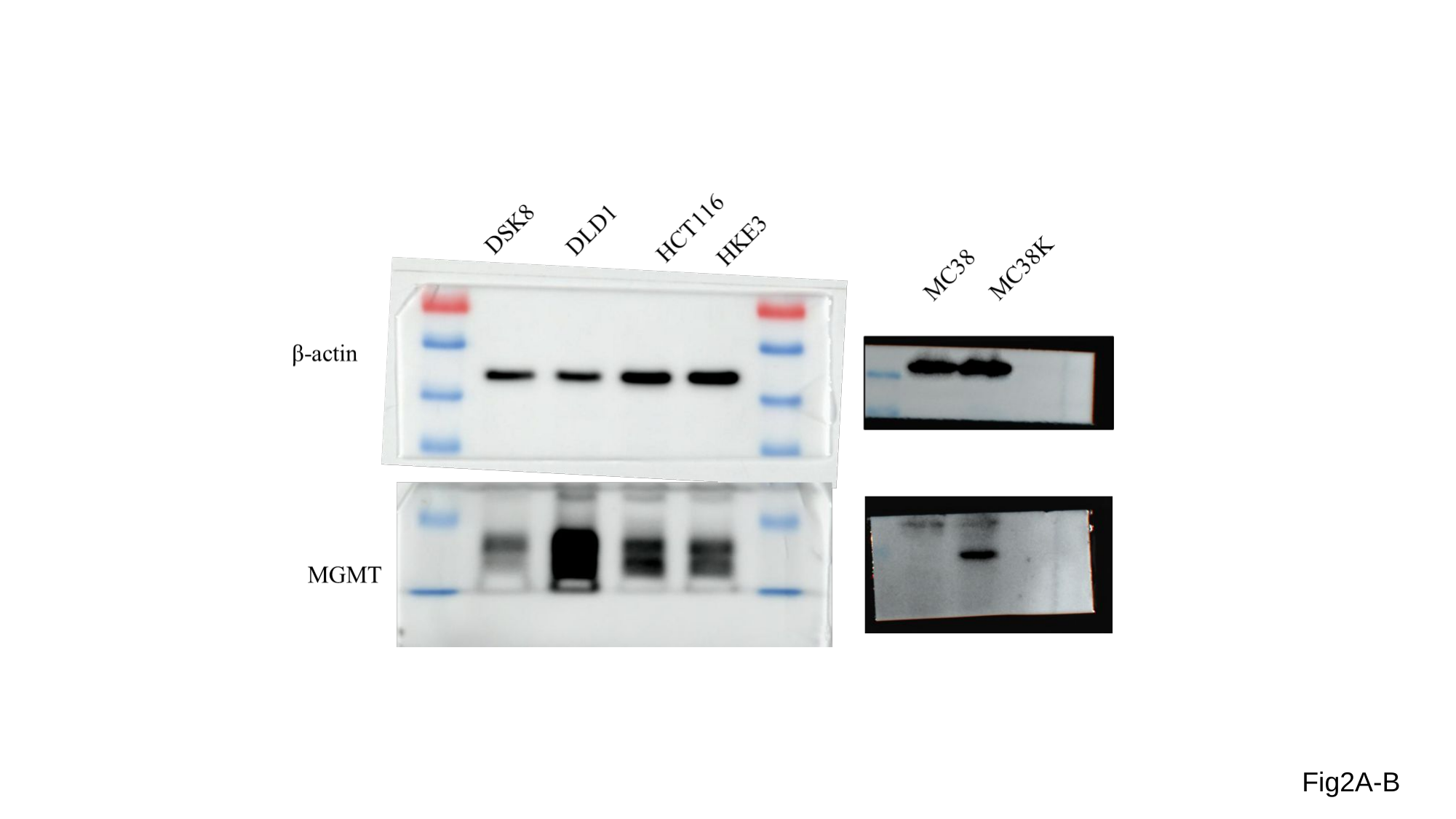

Fig2A-B

## Slide 4
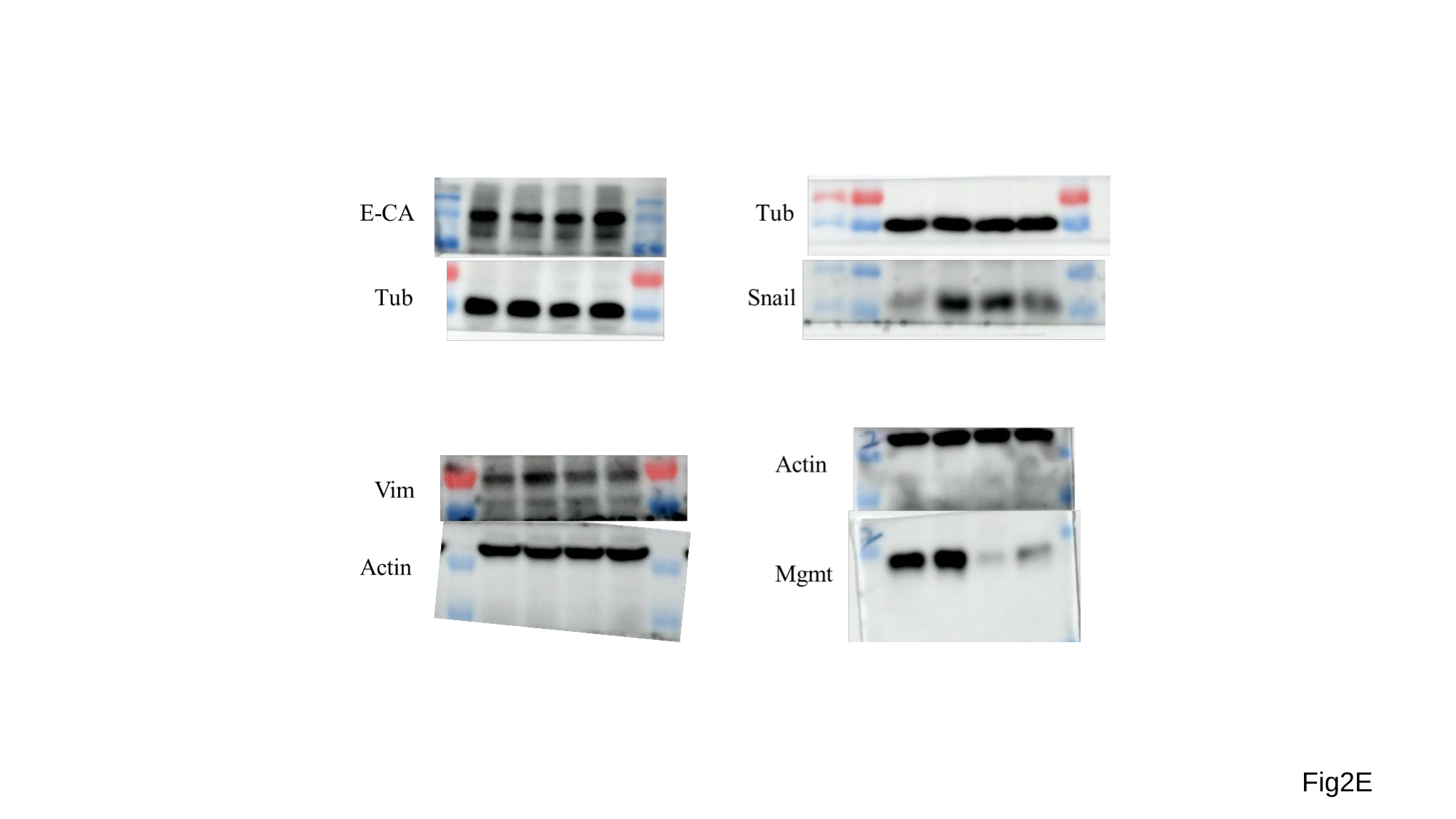

Fig2E

## Slide 5
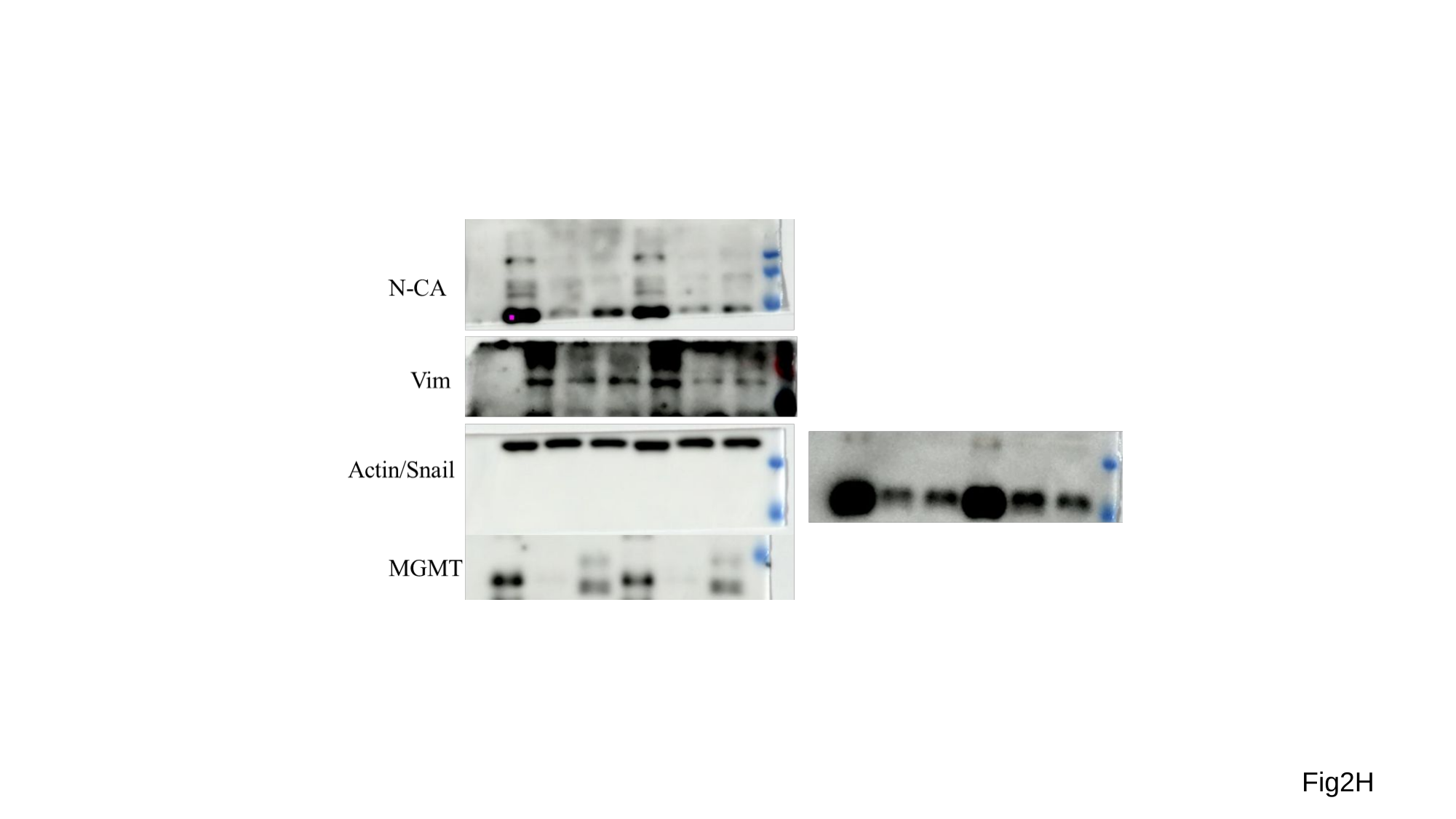

Fig2H

## Slide 6
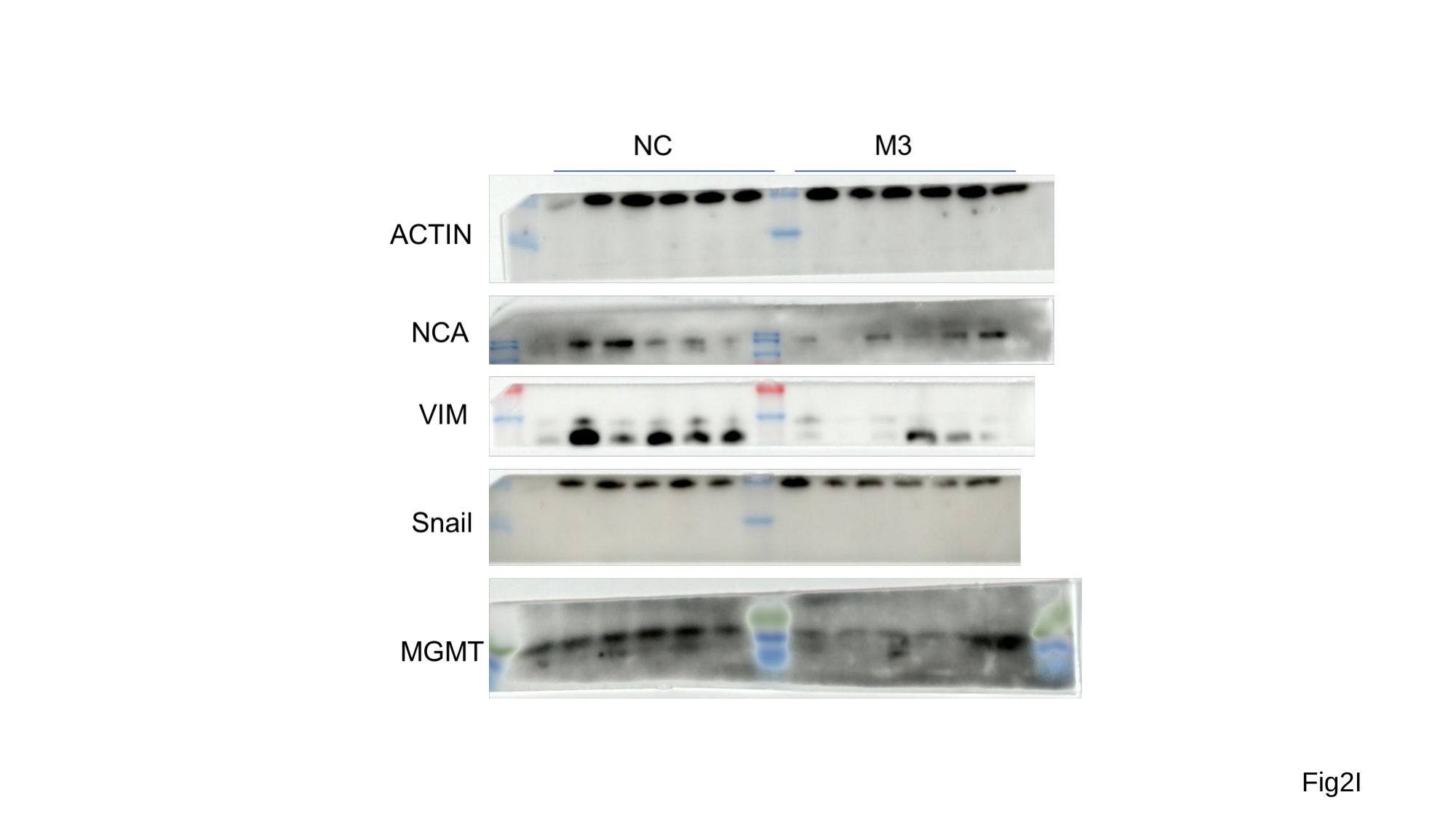

Fig2I

## Slide 7
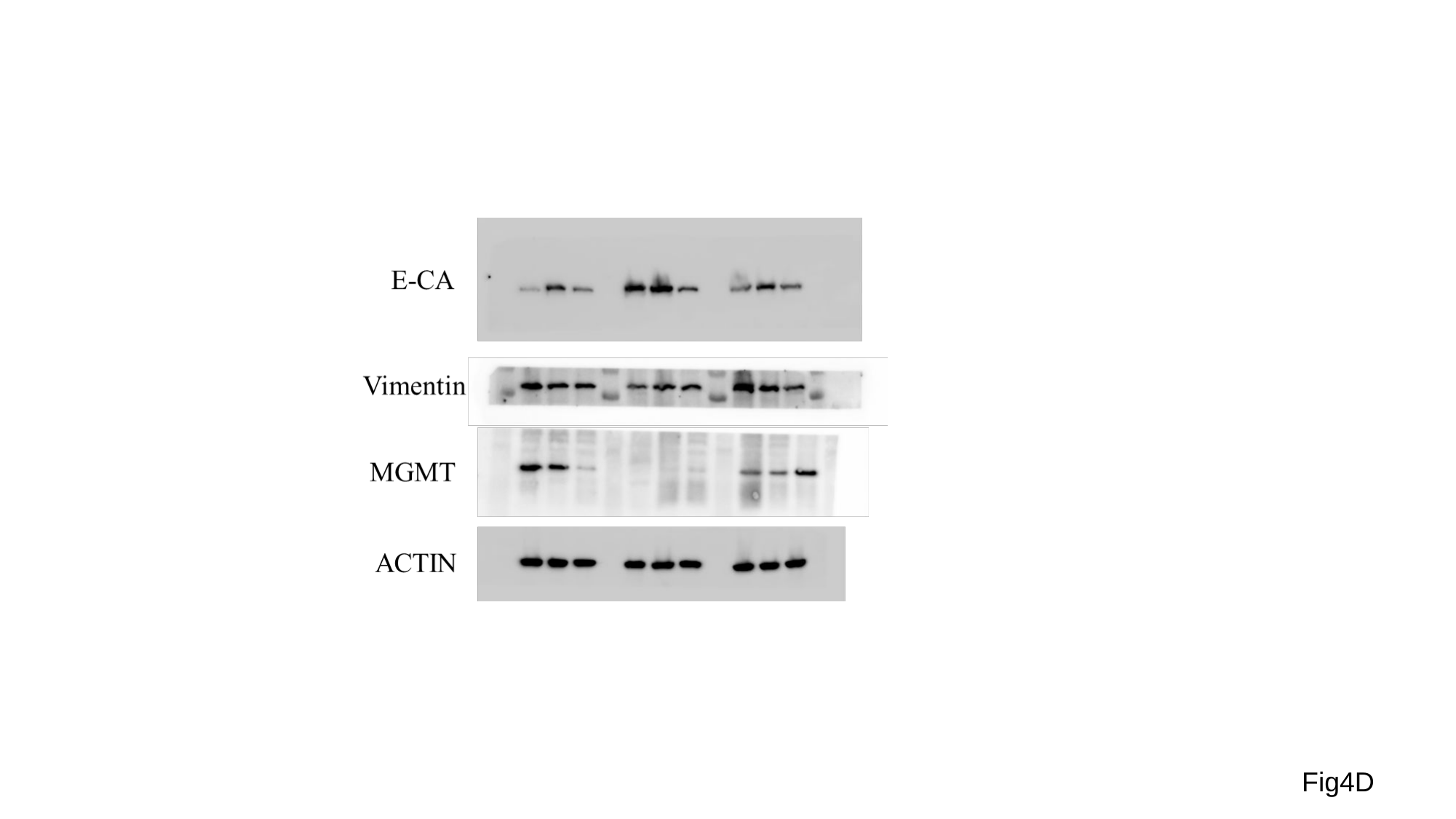

Fig4D

## Slide 8
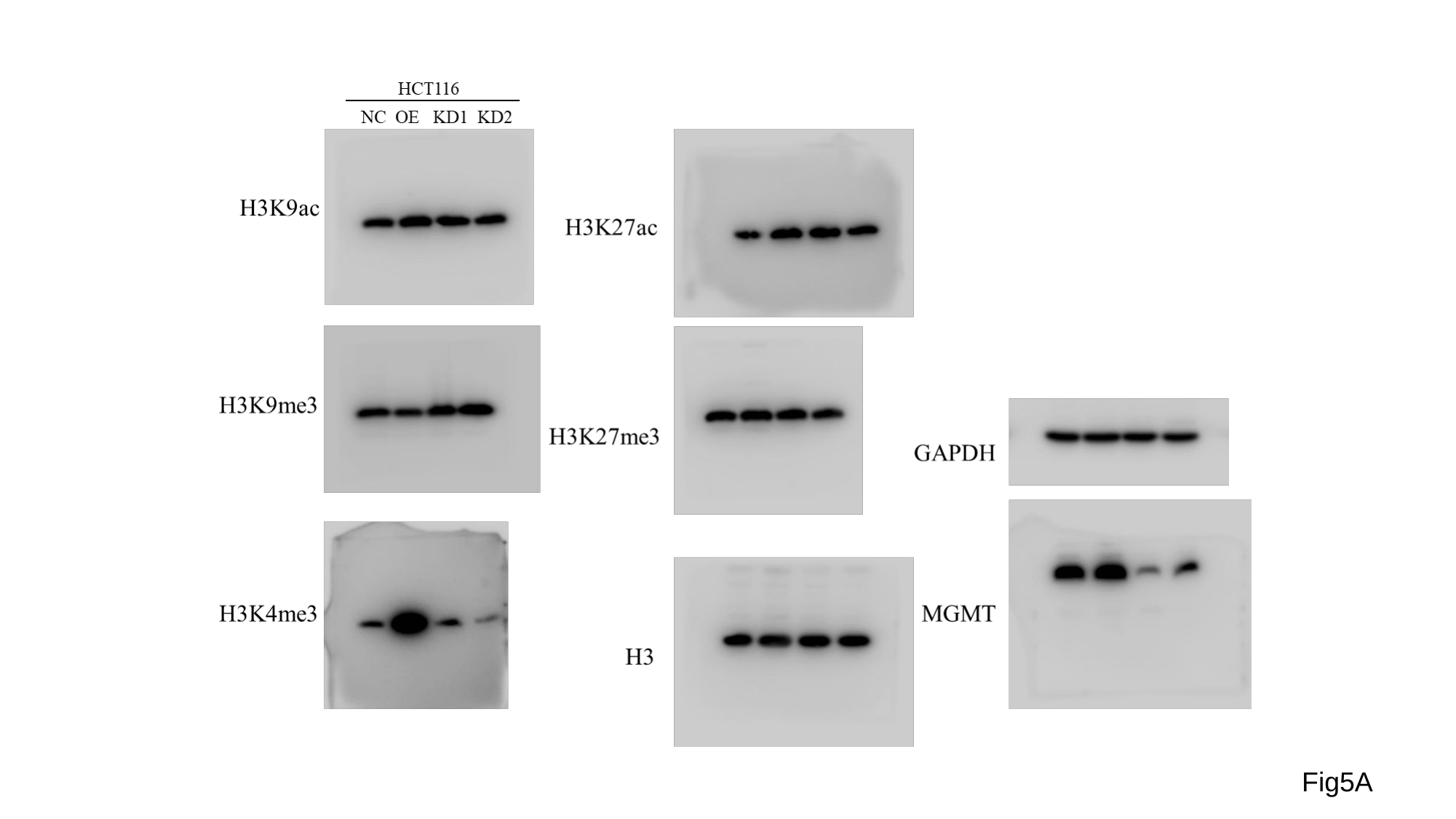

Fig5A

## Slide 9
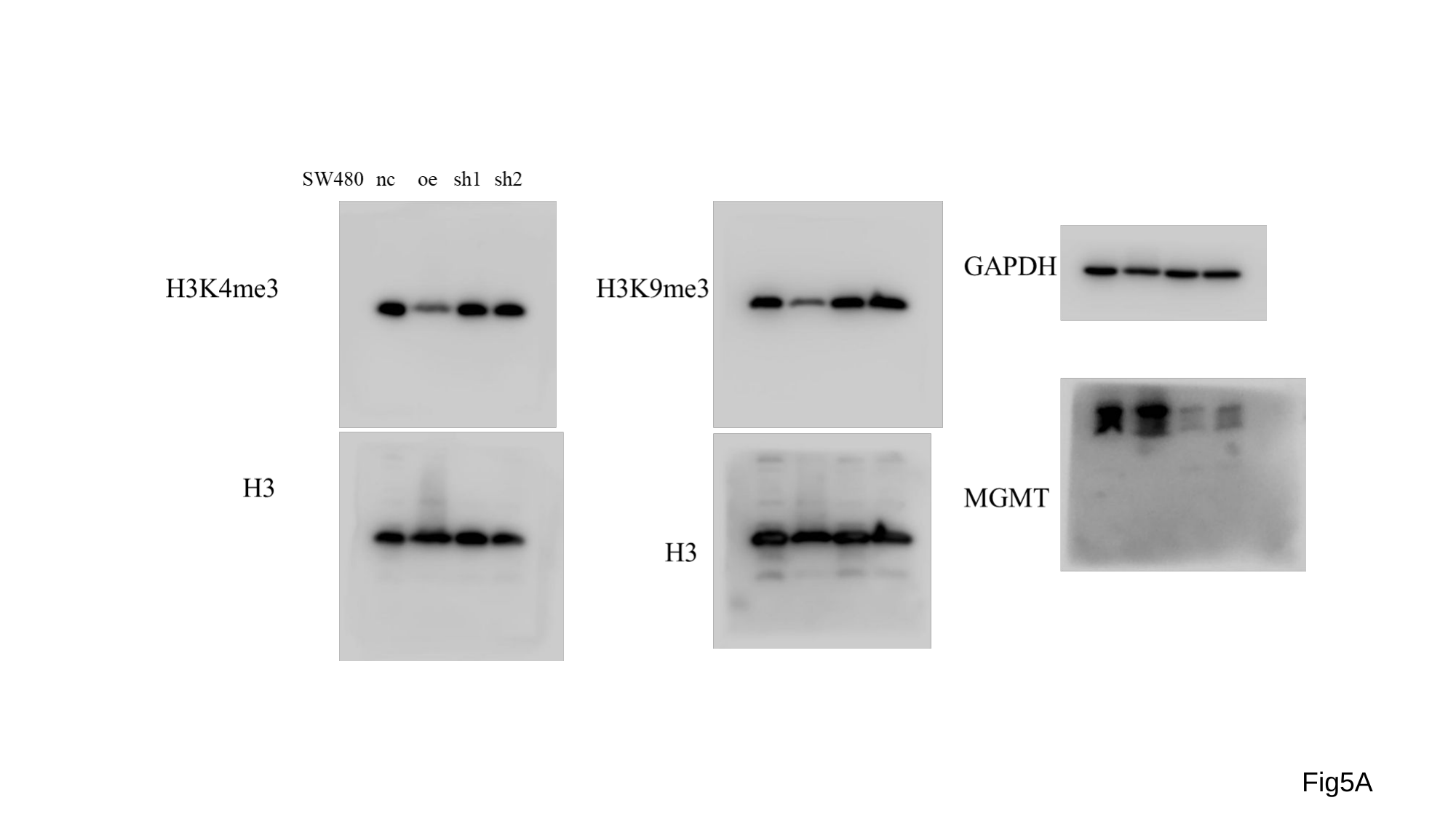

Fig5A

## Slide 10
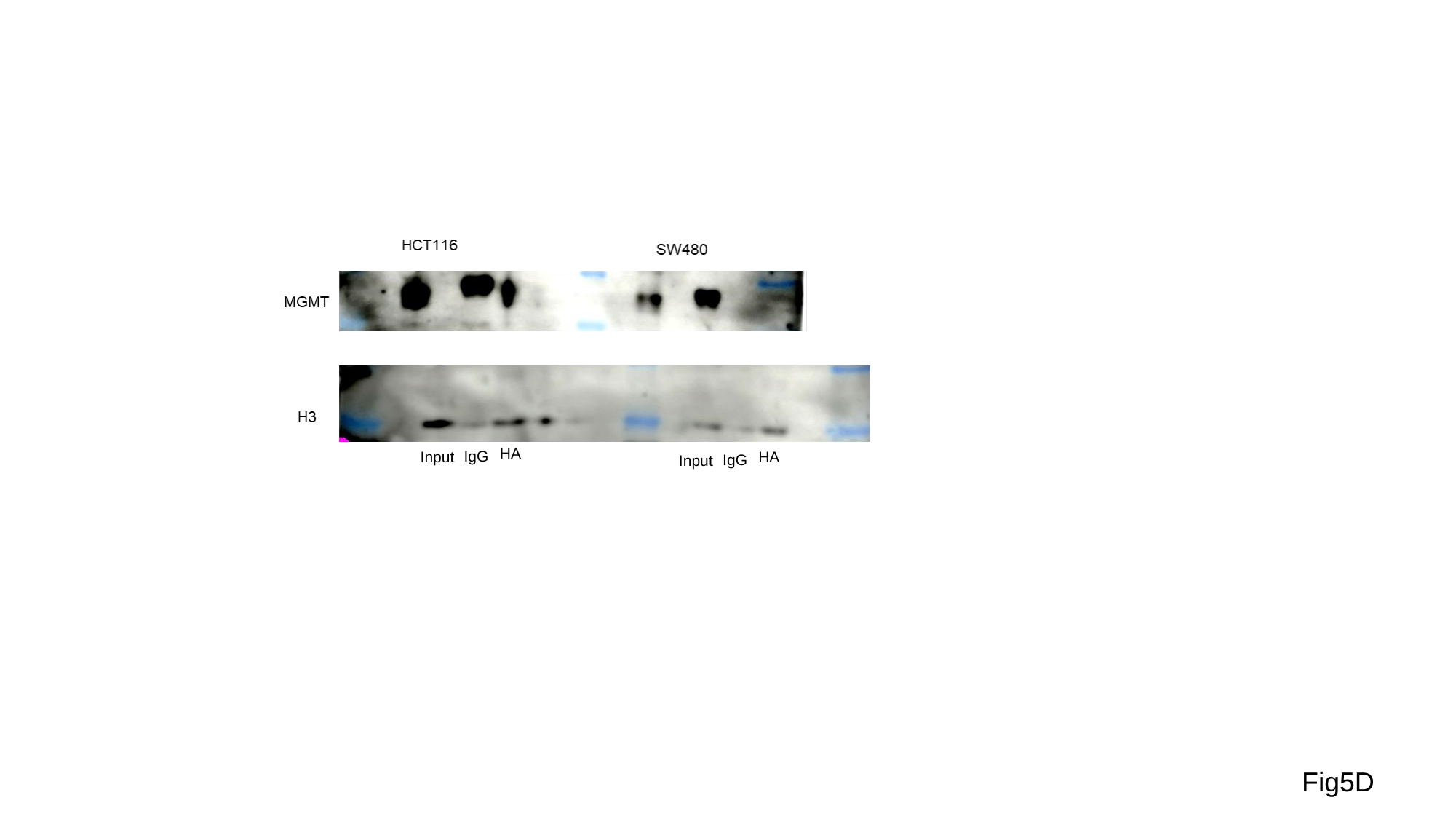

HA
IgG
HA
Input
IgG
Input
Fig5D

## Slide 11
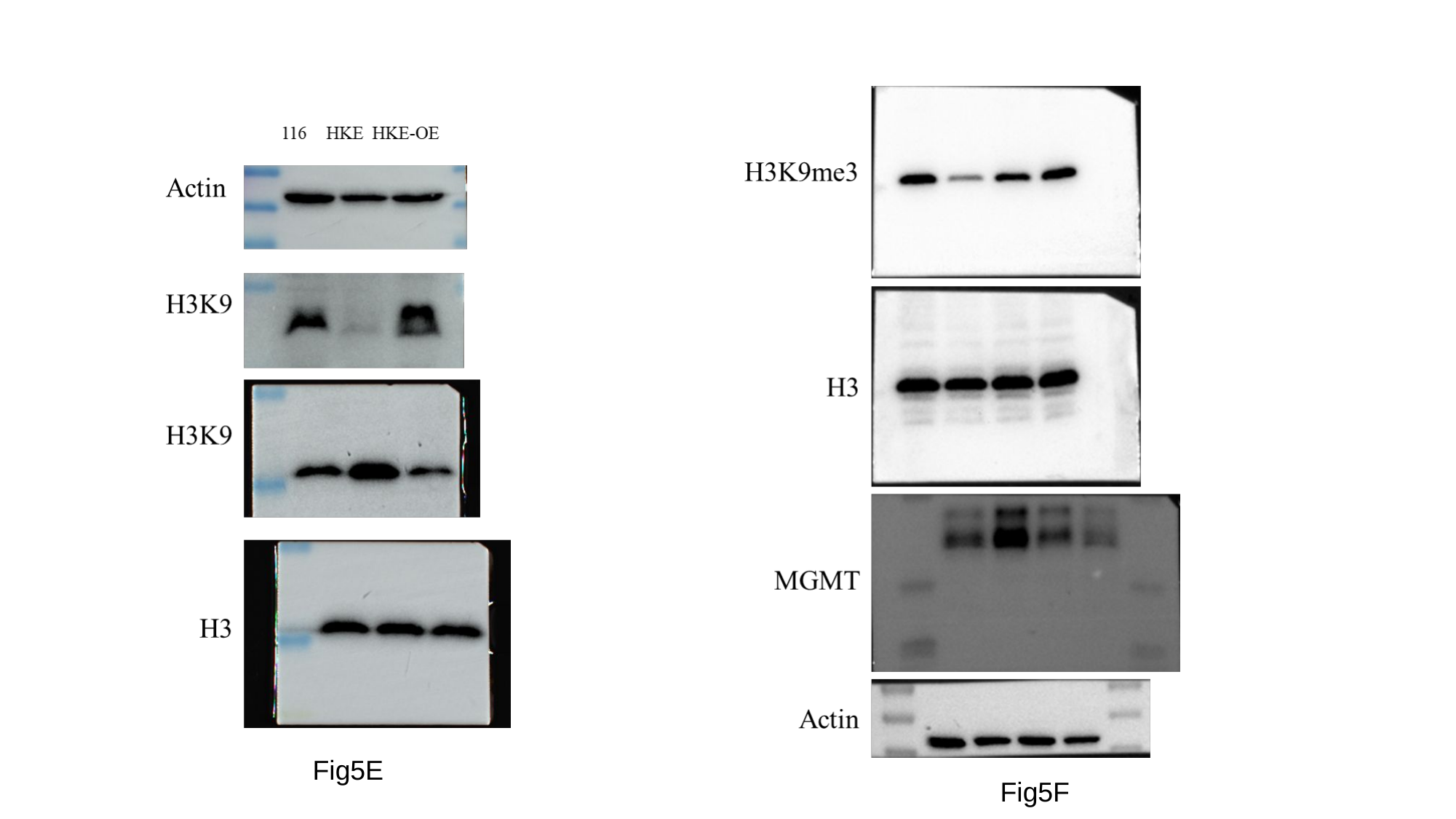

Fig5E
Fig5F

## Slide 12
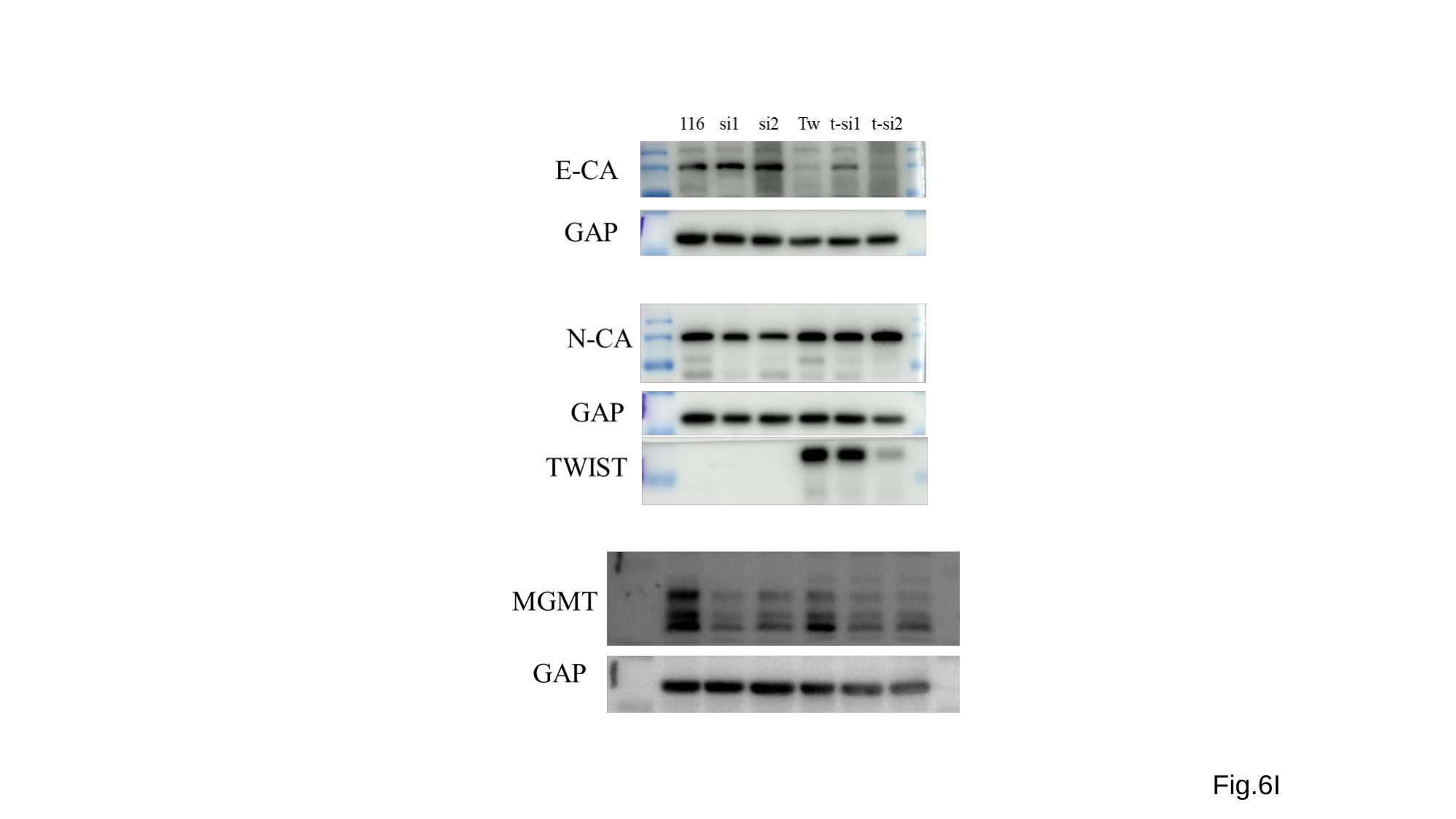

Fig.6I

## Slide 13
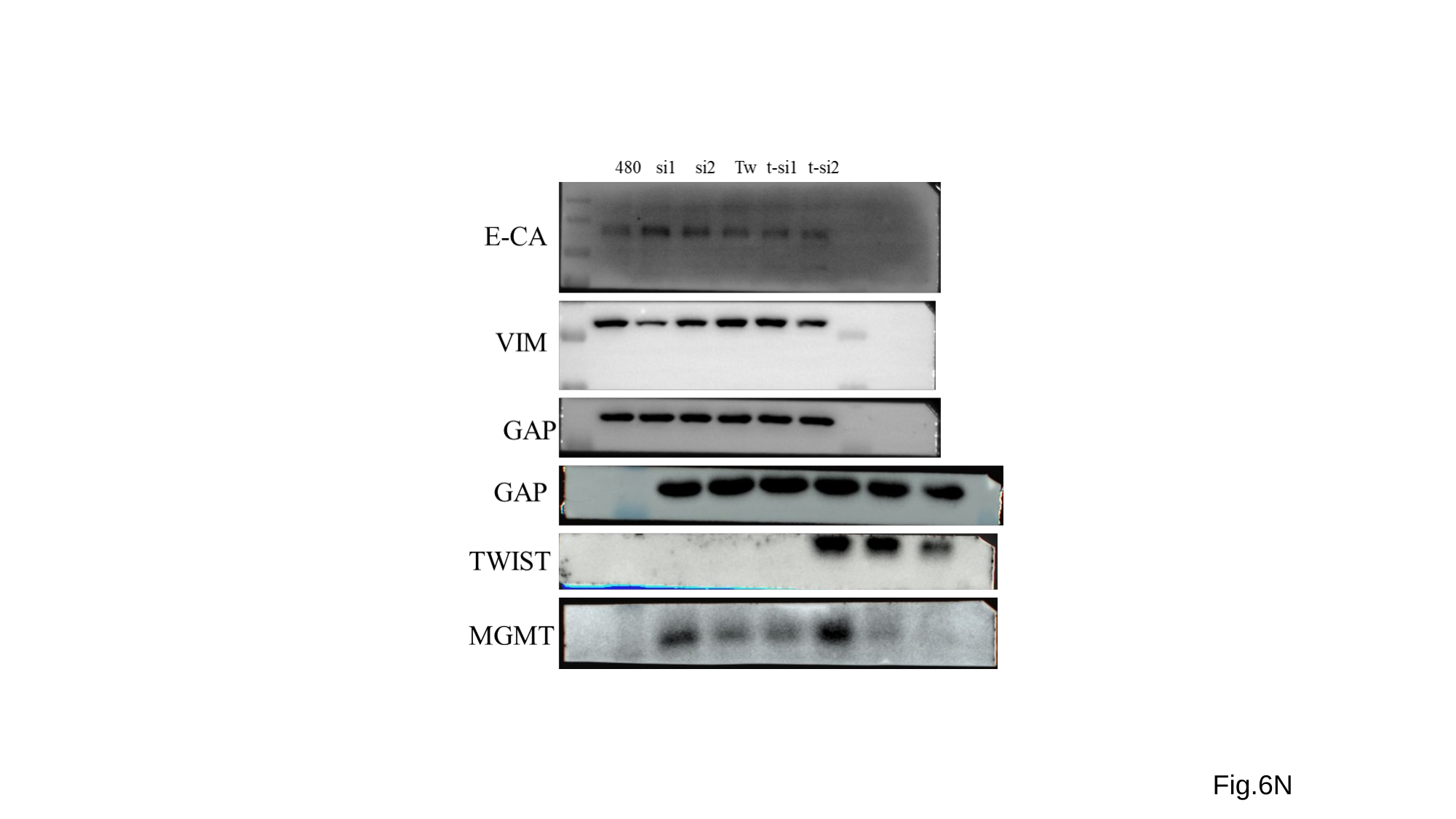

Fig.6N

## Slide 14
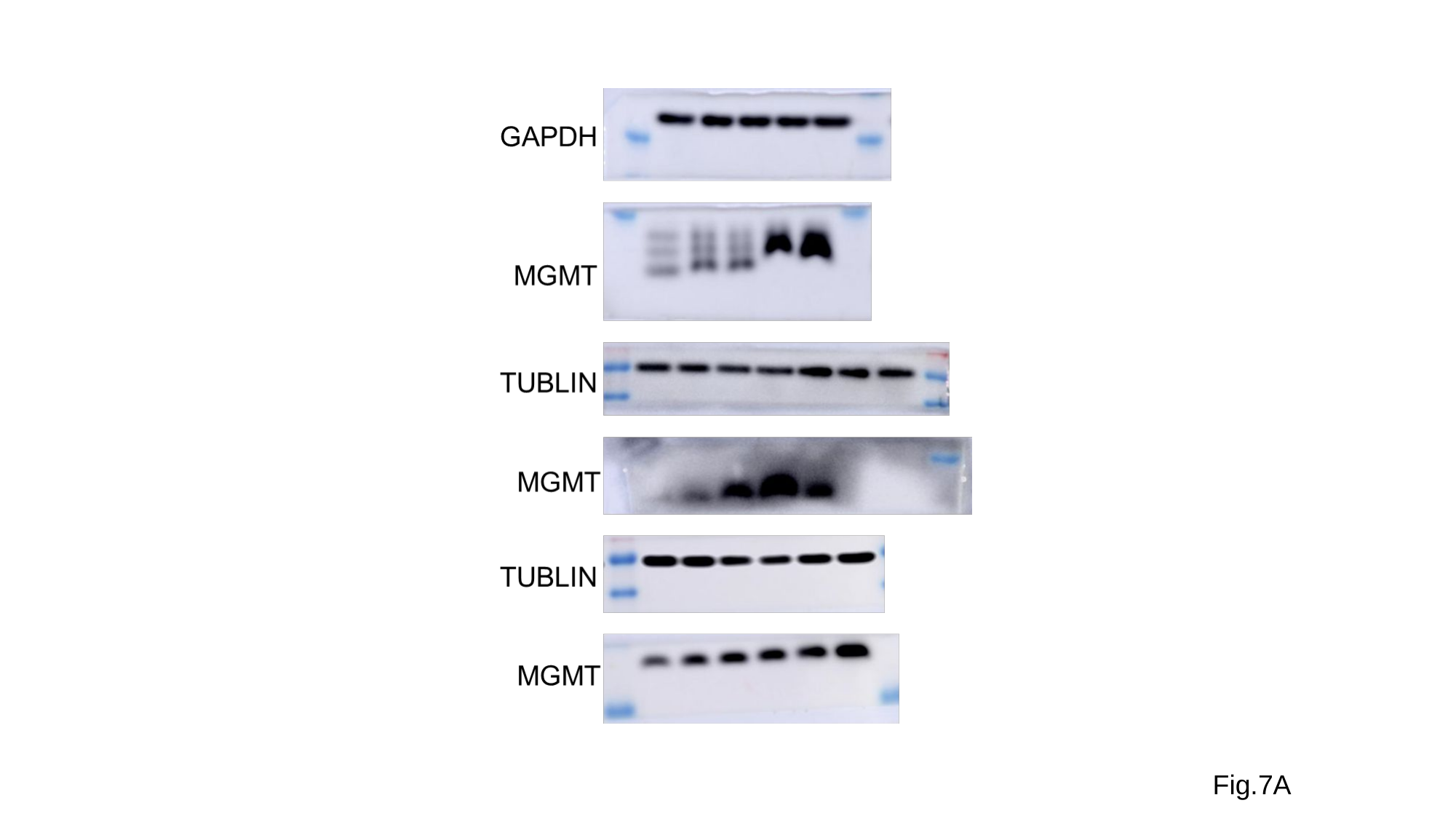

Fig.7A

## Slide 15
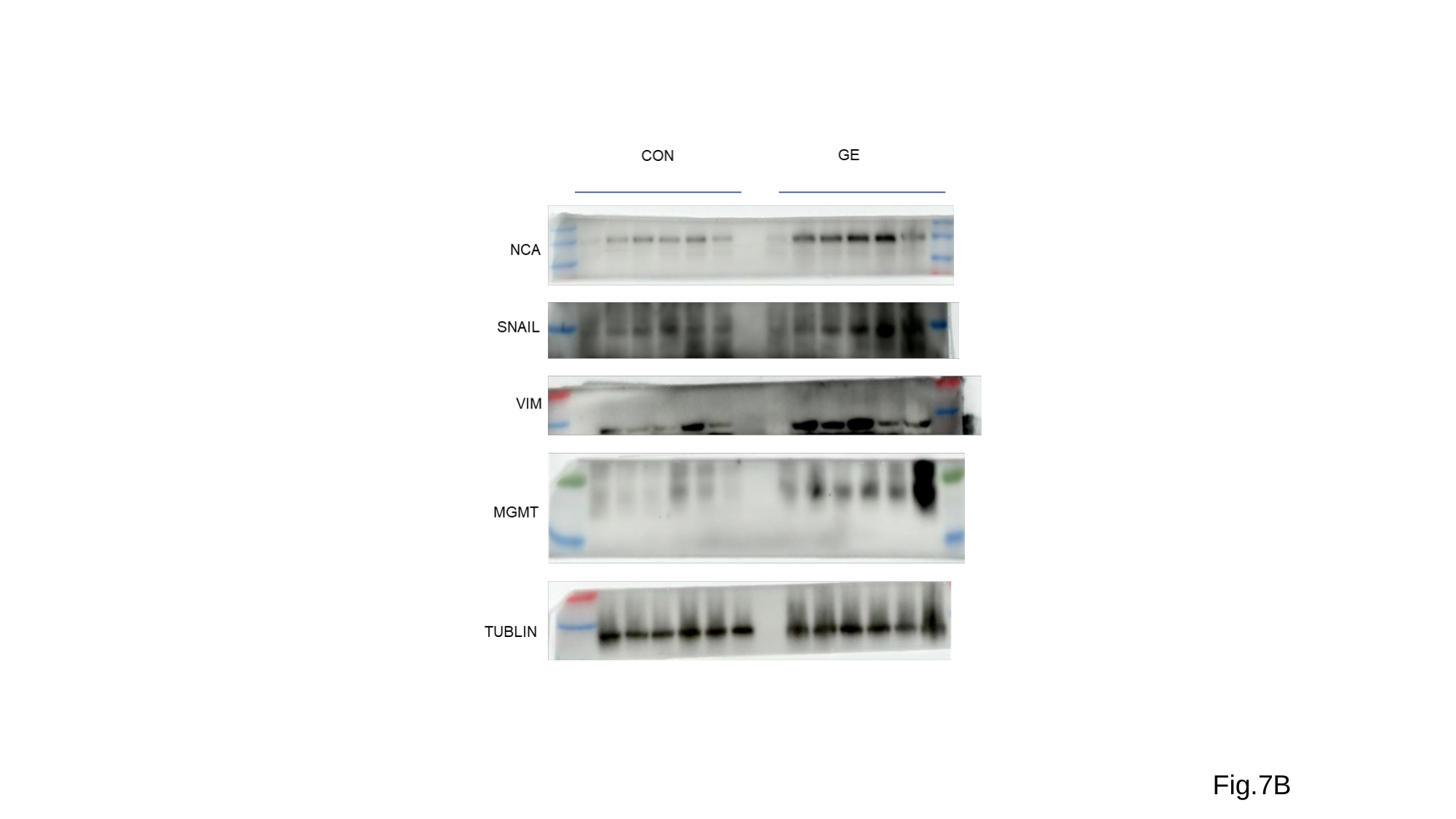

Fig.7B

## Slide 16
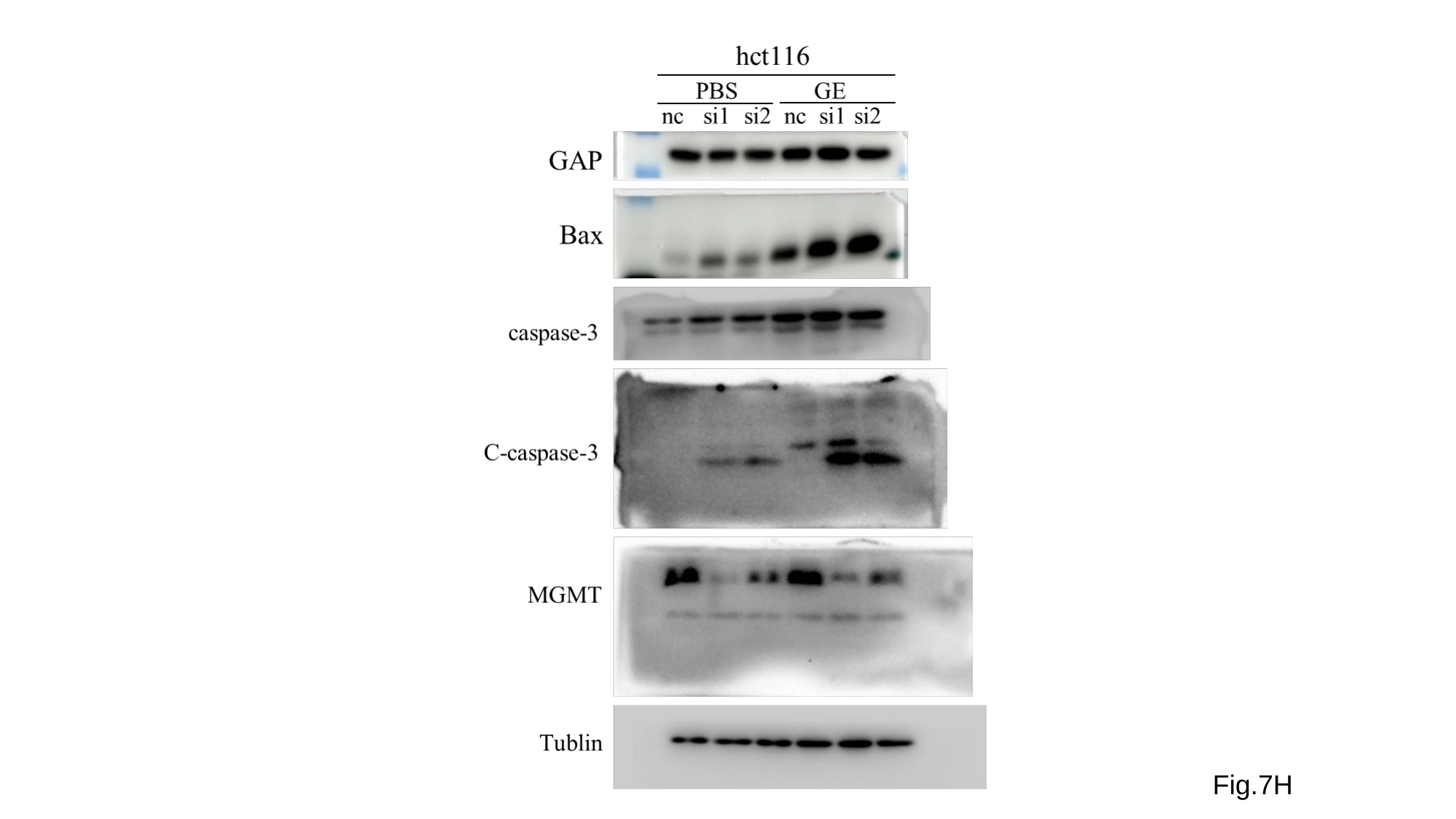

Fig.7H

## Slide 17
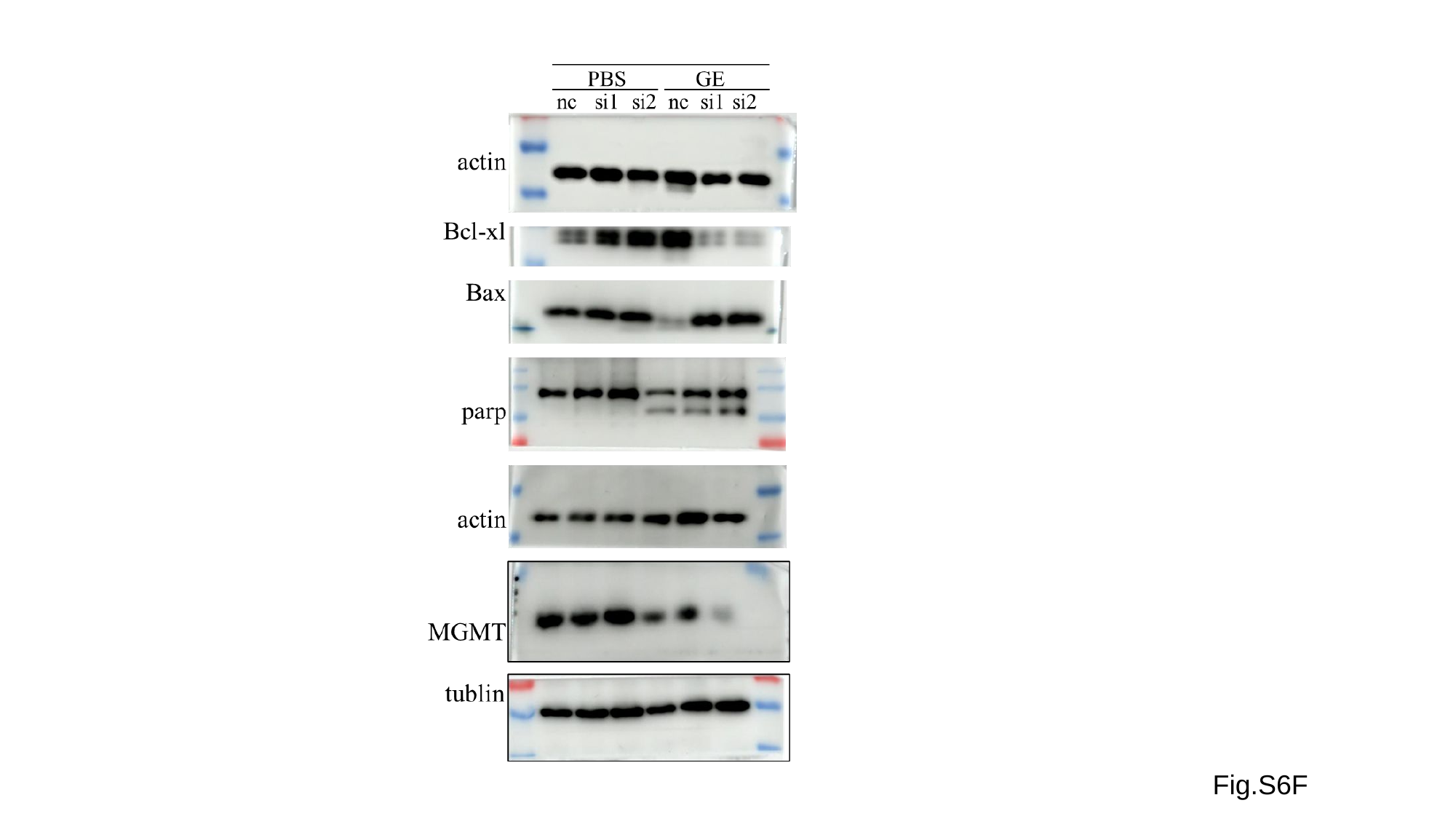

Fig.S6F

## Slide 18
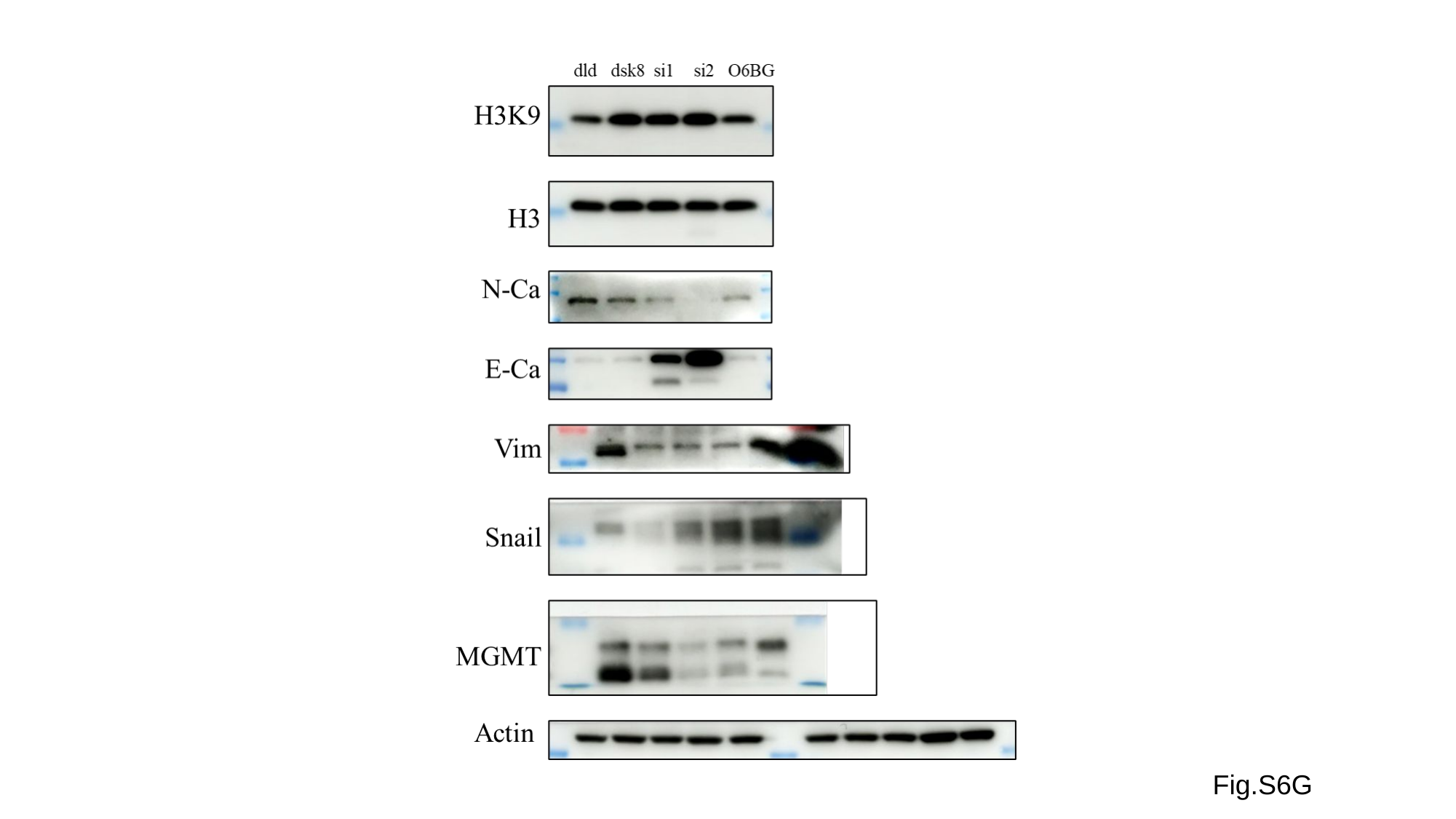

Fig.S6G
